# Same but different — pseudo‐pectin in the charophytic alga *Chlorokybus atmophyticus*

**DOI:** 10.1111/ppl.14079

**Published:** 2023-11-15

**Authors:** Marie N. Rapin, Lorna Murray, Ian H. Sadler, John H. Bothwell, Stephen C. Fry

**Affiliations:** ^1^ The Edinburgh Cell Wall Group Institute of Molecular Plant Sciences, The University of Edinburgh, Daniel Rutherford Building, The King's Buildings Edinburgh UK; ^2^ EastChem School of Chemistry, The University of Edinburgh Edinburgh UK; ^3^ Department of Biosciences Durham University Durham UK

## Abstract

All land‐plant cell walls possess hemicelluloses, cellulose and anionic pectin. The walls of their cousins, the charophytic algae, exhibit some similarities to land plants’ but also major differences. Charophyte ‘pectins’ are extractable by conventional land‐plant methods, although they differ significantly in composition. Here, we explore ‘pectins’ of an early‐diverging charophyte, *Chlorokybus atmophyticus*, characterising the anionic polysaccharides that may be comparable to ‘pectins’ in other streptophytes. *Chlorokybus* ‘pectin’ was anionic and upon acid hydrolysis gave GlcA, GalA and sulphate, plus neutral sugars (Ara≈Glc>Gal>Xyl); Rha was undetectable. Most Gal was the l‐enantiomer. A relatively acid‐resistant disaccharide was characterised as β‐d‐GlcA‐(1→4)‐l‐Gal. Two *Chlorokybus* ‘pectin’ fractions, separable by anion‐exchange chromatography, had similar sugar compositions but different sulphate‐ester contents. No sugars were released from *Chlorokybus* ‘pectin’ by several endo‐hydrolases [(1,5)‐α‐l‐arabinanase, (1,4)‐β‐d‐galactanase, (1,4)‐β‐d‐xylanase, endo‐polygalacturonase] and exo‐hydrolases [α‐ and β‐d‐galactosidases, α‐(1,6)‐d‐xylosidase]. ‘Driselase’, which hydrolyses most land‐plant cell wall polysaccharides to mono‐ and disaccharides, released no sugars except traces of starch‐derived Glc. Thus, the Ara, Gal, Xyl and GalA of *Chlorokybus* ‘pectin’ were not non‐reducing termini with configurations familiar from land‐plant polysaccharides (α‐l‐Ara*f*, α‐ and β‐d‐Gal*p*, α‐ and β‐d‐Xyl*p* and α‐d‐Gal*p*A), nor mid‐chain residues of α‐(1→5)‐l‐arabinan, β‐(1→4)‐d‐galactan, β‐(1→4)‐d‐xylan or α‐(1→4)‐d‐galacturonan. In conclusion, *Chlorokybus* possesses anionic ‘pectic’ polysaccharides, possibly fulfilling pectic roles but differing fundamentally from land‐plant pectin. Thus, the evolution of land‐plant pectin since the last common ancestor of *Chlorokybus* and land plants is a long and meandering path involving loss of sulphate, most l‐Gal and most d‐GlcA; re‐configuration of Ara, Xyl and GalA; and gain of Rha.

## 1 INTRODUCTION

**FIG. 1:**
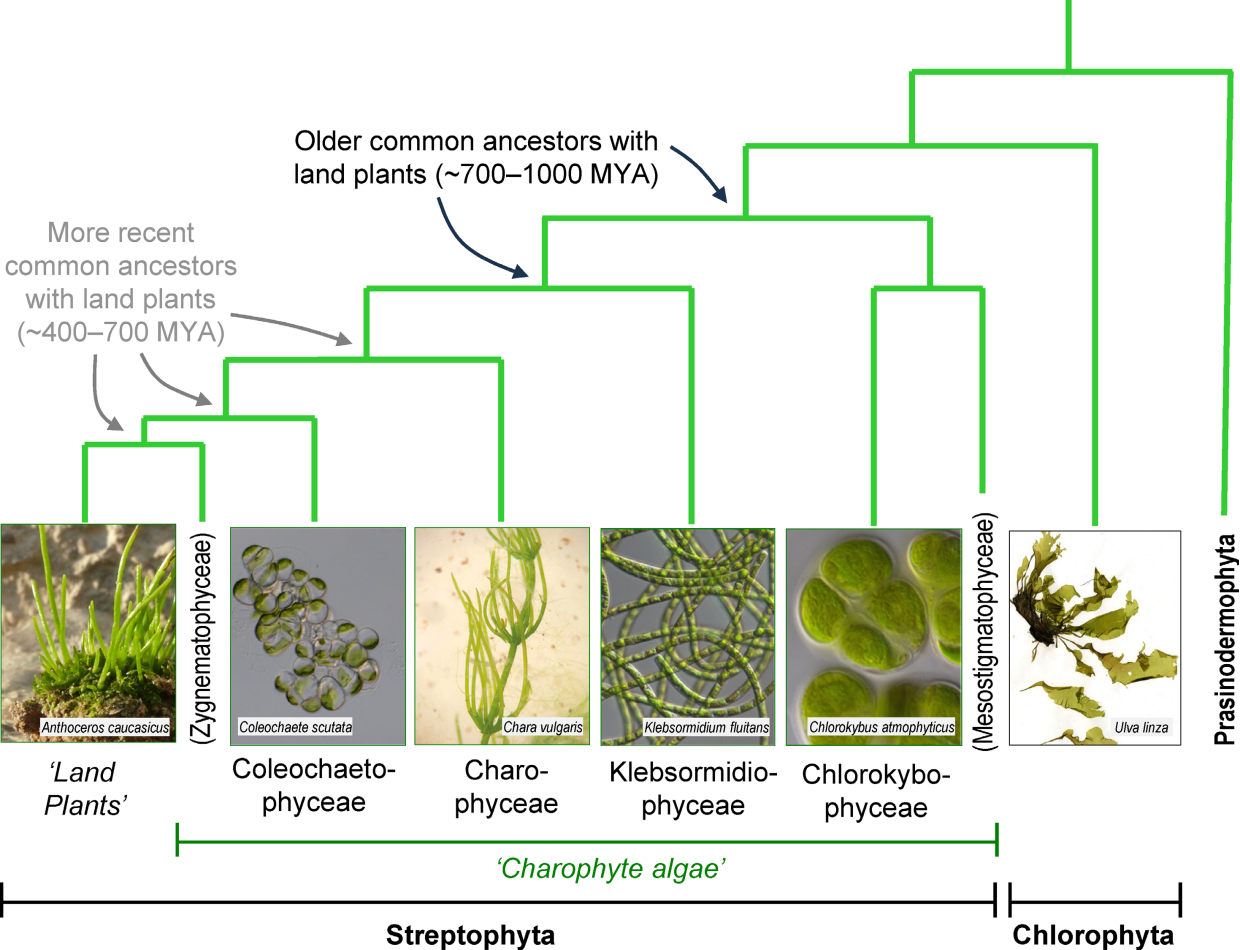
The green plant lineage. The images show the six species studied in this paper. The names of relevant streptophyte classes are shown, with relevant phyla in bold text and less formal names in italics. Classes and phyla that are not studied in this paper are written vertically. The tree is after Li et al. (2020), with broadly indicative dates from Leliaert et al. (2011). Photo credits: *Ulva*, Gabriele Kothe‐Heinrich; Anthoceros, H. Bernd; *Chlorokybus*, Iker Irisarri; *Coleochaete* and *Klebsormidium*, CCAP Culture Collection; *Chara*, Cambridge University Botanic Garden.

Charophytes are, with land plants, part of the Streptophyta (Fig. 1). They are green algae, living in freshwater and in a few cases terrestrial environments. Their pivotal evolutionary position makes them critical for an understanding of the phenomena leading to the birth of the planet's current vegetation. They can be broadly divided into two categories: the early‐ and late‐diverging charophytes (Sørensen et al., 2011). The late‐diverging charophytic algae (classes Zygnematophyceae, Coleochaetophyceae and Charophyceae) present considerable features in common with land plants. The early‐diverging ones (classes Mesostigmatophyceae, Chlorokybophyceae and Klebsormidiophyceae) exhibit more differences from land plants.

The classes of the charophytes that share the most distant common ancestors with land plants are both monogeneric, containing *Mesostigma* and *Chlorokybus* respectively (Fig. 1). *Chlorokybus* has five known species, which, however, are morphologically indistinguishable (Irisarri et al., 2021). *Chlorokybus* spp. form small colonies (sarcinoid thalli) of two or four cells that are embedded in mucilage. They live in moist terrestrial habitats (Cracraft and Donoghue, 2004; Necchi, 2016; Cook and Graham, 2017). The thalli possess a somewhat land‐plant‐like cell wall, composed mainly of polysaccharides, though including only small amounts of cellulose (Kiemle, 2010; Sørensen et al., 2011; O'Rourke et al., 2015). It is interesting that there are many more differences in cell wall composition amongst the chlorophytes than there are amongst the streptophytes (charophytes plus land plants) (Kirkwood, 1974). However, most of the *Chlorokybus* polysaccharides are clearly distinct from those found in land plants and are still poorly characterised.

Across all the streptophytes, hemicelluloses and pectin are the main non‐cellulosic polysaccharides of the cell wall matrix. In general, the hemicelluloses are uncharged or mildly anionic. They are not extractable from cell walls in water but are extractable in concentrated aqueous alkali (Scheller and Ulvskov, 2010), ideally 6 M NaOH at 37°C (Edelmann and Fry, 1992). Once extracted in NaOH, most hemicelluloses remain in solution upon neutralisation and can hydrogen‐bond to cellulose. In the much‐studied primary cell walls of land plants, the major hemicelluloses are xyloglucan, (hetero)xylans, (hetero)mannans and in some taxa mixed‐linkage β‐glucans. In charophytic algal cell walls, xyloglucan is either absent (Popper & Fry, 2003) or present in only small quantities (Mikkelsen et al., 2021). Xylans, including arabinoxylans, are present in many charophytes (Popper and Fry, 2003; Jensen et al., 2018). Abundant mannose occurs in charophyte cell wall hydrolysates, suggesting the presence of mannans (Popper and Fry, 2003; Scheller and Ulvskov, 2010). Mannan has also been evidenced immunologically in *Chara* (Domozych et al., 2009, 2010) and across the charophytes, though in variable quantities (Sørensen et al., 2011). In the more basal streptophyte algae, the picture is less clear. In *Chlorokybus*, 4‐linked xylose residues were detected in the hemicellulose fraction, which might be indicative of xylans (Kiemle, 2010). Mixed‐linkage glucans (MLGs) were evidenced immunologically in some species of the Zygnematophyceae (Domozych et al., 2012). MLGs were not found in species of the Charophyceae and Coleochaetophyceae when examined by enzymic methods (Popper and Fry, 2003; Sørensen et al., 2011), but were characterised by enzymic methods coupled with mass‐spectrometry in some of the Klebsormidiophyceae, Chlorokybophyceae, Charophyceae and Zygnematophyceae (Kiemle, 2010).

In contrast to hemicelluloses, pectic domains are highly anionic and do not usually hydrogen‐bond to cellulose. Of the pectic domains found in land plants, the simplest and the only one so far definitively detected in charophytes is homogalacturonan, a linear polysaccharide based on (1→4)‐α‐galacturonan. Galacturonic acid (GalA) was detected in acid‐hydrolysates of all charophyte cell walls examined except *Klebsormidium*, sometimes in higher concentrations than in land plants (Popper and Fry, 2003). All charophytes that yielded GalA on acid hydrolysis, including *Chlorokybus*, also yielded it on digestion with Driselase (O'Rourke et al., 2015), an enzyme mixture produced for digestion of land‐plant cell walls, suggesting that the GalA‐containing polymer of charophytes was classic homogalacturonan. Homogalacturonan was also evidenced immunologically across many charophyte taxa (Zygnematophyceae, Coleochaetophyceae, Charophyceae), mostly with little or no methyl‐esterification (Domozych et al., 2010; Sørensen et al., 2011; Herburger et al., 2019). Its presence, as well as evidence for cross‐linking with Ca^2+^, was confirmed by more specific studies in the Charophyceae and Zygnematophyceae (Cherno et al., 1976; Domozych et al., 2007, 2010; Eder and Lütz‐Meindl, 2008, 2010; Domozych et al., 2009; Anderson, 2016). However, homogalacturonan was undetectable in all members of the Klebsormidiophyceae studied (O'Rourke et al., 2015), agreeing with the absence of GalA (Popper & Fry, 2003), so it seems to have been lost during the evolution of this class.

A relatively unfamiliar ‘pectic’ sugar residue, 3‐*O*‐methyl‐l‐rhamnose (acofriose) was found in both early‐ and late‐diverging charophytes, as well as in bryophyte cell walls. This sugar was undetectable in later‐diverging (vascular) land plants (Popper et al., 2004). In the same way, 3‐*O*‐methyl‐d‐galactose residues in pectic extracts have been found in the late‐diverging charophytes, *Chara* and *Coleochaete*, and it appears that this sugar may have been replaced by arabinose in land plants (O'Rourke et al., 2015).

It is evident that the ‘pectic’ domains familiar from studies of land plants (homogalacturonan, rhamnogalacturonan‐I and rhamnogalacturonan‐II) have arisen from a range of highly dissimilar pectic polysaccharide(s) present in charophytes. The major goal of the present work was to characterise the cell wall in one of the earliest‐diverging charophytes, *Chlorokybus*, which shares an evolutionarily distant last common ancestor with land plants, with special reference to the pectin (or pectin‐like) fraction, and to make sense of the ultimate origins of the streptophyte cell wall.

## 2 METHODS

### 2.1 Plants (including algae)

Our culture of *Chlorokybus atmophyticus* was from CCAP (Dunstaffnage, UK). Other cultures were sourced and maintained as described before (Franková and Fry, 2012; O'Rourke et al., 2015). All the charophytes (*Chlorokybus atmophyticus, Klebsormidium fluitans*, *Coleochaete scutata*, and *Chara vulgaris*) and the bryophyte (*Anthoceros caucasicus*) were cultivated in the laboratory, in 100 ml of medium per 250‐ml conical flask, under constant low light at 24°C. *Chlorokybus* was grown in soil medium (JM:SE2, 7:3) (Schlösser 1994). All other charophytes were grown at 25°C on Bold basal medium (3N‐BBM+V) (Bischoff et Bold 1963). *Anthoceros* cell‐suspension cultures (kindly donated by Dr Maike Peterson, Marburg University) were grown as described before (O'Rourke et al., 2015). *Ulva* was wild‐collected at North Berwick, UK, and provisionally identified as *Ulva linza* because of its location, branched morphology and ultrastructure (Brodie et al., 2007).

### 2.2 Preparation of AIR

For isolation of polysaccharide‐rich material, plant samples (~100 mg fresh weight) were stirred in 50 ml 70–77% (v/v) ethanol at 20°C for 16 h, then centrifuged at 5000 *g* for 10 min. The resulting cell wall‐rich alcohol‐insoluble residue (AIR) was washed several times in 70% ethanol, 96% ethanol, then acetone, and finally dried at approx. 20°C.

### 2.3 Amylase digestion

When stated in the Results, AIR samples were de‐starched with α‐amylase. The AIR was suspended at 10 mg.ml^−1^ in 40 mM lutidine (OAc^−^) buffer, pH 6.7, in 0.25% (w/v) chlorobutanol (volatile antimicrobial agent), stirred at 100°C for 15 min (gelatinising any starch), and cooled to 60°C. Next, 0.1 volumes of a solution of heat‐stable α‐amylase [prepared from 10 ml of the commercial solution dialysed against water then diluted to 45 ml with lutidine buffer (40 mM, adjusted with acetate to pH 6.5)] was added, giving a final enzyme concentration of 4 U/ml, and incubation was conducted at 60°C for 72 h. Ethanol and ammonium formate were then added to give final concentrations of 70% (v/v) and 1% (w/v), respectively, and the suspension was incubated at 20°C for 16 h, precipitating any buffer‐solubilised polysaccharides among the cell walls, which were thoroughly rinsed with 70% ethanol and dried. The ethanolic supernatant was also kept for malto‐oligosaccharide analysis.

### 2.4 Endo‐polygalacturonase (EPG) digestion of AIR

Where indicated, AIR samples were digested with EPG. The AIR (2.5 mg.ml^−1^) was de‐esterified in 1 M NaOH in 75% ethanol for 10 min at 20°C, then centrifuged at 4000 *g* for 5 min. The pellet was washed in 75% ethanol for 10–20 minutes, 100% ethanol for 10–20 minutes, ethanol containing 0.5% acetic acid, then 100% acetone, and dried in a SpeedVac. The AIR (20 mg.ml^−1^) was then digested with EPG (Megazyme; 2.5 U.ml^−1^) in pyridine/acetic acid/water 1:1:98, pH 4.7, at 20°C for 16 h with gentle shaking. Water (0.5 vol.) was added, the mixture was centrifuged and the clear supernatant was collected. The digestion was repeated on the insoluble residue and the second supernatant pooled with the first.

### 2.5 Enzymes and chemicals

Enzymes used on algal extracts are listed in Table S1. All chemical reagents were purchased from Sigma Aldrich, Fisher Scientific, and VWR. All water used was deionised water unless otherwise stated. Silica TLC plates (plastic and aluminium‐backed) were sourced from Merck.

### 2.6 Differential solubilisation of polysaccharide classes from AIR

Polysaccharide fractions were prepared from the AIR following a sequential extraction process, incubating plant polymers successively in: 0.2 M oxalate [ammonium^+^, pH 4.1, at 100°C for 2 h (giving extract P1) and a further 16 h (extract P2)]; 6 M NaOH at 37°C for 72 h (giving hemicelluloses); and 0.2 M acetate (Na^+^, pH 4.0; 20°C, giving extract W). The final residue was α‐cellulose (αC). After each extraction, the suspension was centrifuged at 5000 *g* for 10 min. The hemicellulose extract was adjusted to pH ~4.2 by addition of acetic acid, then stored overnight at 20°C: the hemicellulose a (Ha) precipitated and was pelleted in a bench centrifuge as above, leaving soluble hemicellulose b as the supernatant. Ha and αC were washed in water. P1, P2, Hb and W were dialysed against deionised water. Finally, all fractions were freeze‐dried.

### 2.7 Acid hydrolysis

For ‘complete’ acid hydrolysis, AIR or polysaccharide fractions (5 mg) were hydrolysed with 1 ml of 2 M trifluoroacetic acid (TFA) at 120°C for 1 h. This procedure is expected to hydrolyse essentially all neutral sugar residues except those of cellulose, and to partially hydrolyse uronosyl linkages (Fry, 2000). Thus the disaccharide Gal→GlcA would be completely hydrolysed, whereas GlcA→Gal would be only partially hydrolysed. For ‘mild’ acid hydrolysis of ion‐exchange chromatography fractions, AIR or polysaccharide fractions (5 mg dry weight) were hydrolysed with 1 ml of 0.1 M trifluoroacetic acid (TFA) at 100°C for 2 h. This procedure is expected to hydrolyse all furanosyl linkages. The hydrolysate was dried *in vacuo*, re‐dissolved in water and chromatographed.

### 2.8 Chromatography

Thin‐layer chromatography (TLC) was usually performed on Merck silica‐gel 60 plastic‐backed plates. For analysis of oligogalacturonides, Merck silica‐gel 60 F_254_ aluminium‐backed plates were used. Solvents were: BAW (butan‐1‐ol/acetic acid/water, 4:1:1 unless otherwise stated) and EPAW (ethyl acetate/pyridine/acetic acid/water, 6:3:1:1). Usually two ascents of the solvent were used, the plate being dried between each ascent. Sugars on TLC plates were stained with thymol/H_2_SO_4_ (Jork et al., 1994).

Paper chromatography was performed by the descending method on Whatman paper No. 1 when used for analytical purposes and No. 3 when used for preparative purposes, in butan‐1‐ol/acetic acid/water (12:3:5, v/v/v) for 24 h. For preparative paper chromatography (PPC), markers and part of the sample were stained with aniline hydrogen‐phthalate (Fry 2000). The portions of interest (unstained) were then cut off the sheet and sugars eluted with 75% ethanol by the method of Eshdat & Mirelman (1972).

*Chlorokybus* ‘pectin’ (5 mg) was fractionated on a 10‐ml Q‐Sepharose column, which comprises cross‐linked 6% agarose beads with quaternary ammonium (strong anion‐exchange) groups. Samples were eluted at ~30 ml/h with a range of pyridinium acetate buffers (11–1400 mM pyridine) adjusted to pH 5.3 with acetic acid and containing 8 M urea, followed by a 2 M acetate (Na^+^) buffer, pH 7.0, also containing 8 M urea, and two successive alkali solutions at 1 M and 6 M NaOH (Popper and Fry 2005). Each buffer was applied as two 10‐ml portions, the two eluates being collected separately. The urea was added as a precaution to minimise the hydrogen‐bonding of polysaccharides to each other and to the column matrix. Prior to all subsequent analyses, the urea was removed by dialysis.

### 2.9 Dot‐blot thymol assay

For approximate assays of total carbohydrate, a 1–3‐μl sample solution was loaded as a dot on a TLC plate, alongside a dilution series of the appropriate reference sugar (glucose, galacturonic acid, etc.). The plate was air‐dried for at least 30 minutes, then stained with thymol/H_2_SO_4_ (Jork et al., 1994). The intensity of staining was measured with ImageJ, and a reference graph of staining intensity against concentration was created. The sugar concentration of the sample solution was calculated by linear regression.

### 2.10 Uronic acid assay

Uronic acids were measured in aqueous solutions by the *m*‐hydroxybiphenyl assay of Blumenkrantz & Asboe‐Hansen (1973).

### 2.11 Sulphate assay

Prior to sulphate assay, polymer samples were fully hydrolysed (2 M TFA, 120°C, 1 h), dried, re‐dissolved in water, re‐dried, and re‐dissolved in 1 M acetic acid such that the sugar concentration was 0.3 mg.ml^‐1^. The absorbance at 450 nm was measured. Saturated barium acetate was added to the solution to give 0.15 M barium acetate. The solution was thoroughly mixed, producing a turbid suspension of barium sulphate, which was quantified from the new absorbance at 450 nm. The method was calibrated with a series of standards of sodium sulphate, 2–128 mM.

### 2.12 NMR spectroscopy

1‐D and 2‐D ^1^H and ^13^C NMR spectra were recorded on a Bruker AVANCE NEO instrument (18.8 T; 800 MHz for protons) with D_2_O as solvent. Chemical shifts (Table 1) are given in ppm (δ), and scalar coupling constants (*J*) are given in Hz.

**TABLE 1:** ^1^H‐ and ^13^C‐NMR spectral data of aldobiouronic acid A1 from *Chlorokybus* pectin [β‐d‐glucuronosyl‐(1→4)‐l‐galactose].

Site	δ_c_	δ_H_	Multiplicity	J_HH_ (Hz)	Proton–carbon long‐range correlation (from HMBC spectrum)
*α‐Galactose (residue A)*					
A1	92.3	5.219	*(d)*	3.3	
A2	68.5	3.776	*(dd)*	3.4. 10.0	
A3	68.5	3.44–3.46	*(dd)*	2.6, 10.0	
A4	78.8	4.126	*(d)*	2.6	A4/C1
A5	70.5	4.110	*(t)*	6.3	
A6a, A6b	60.2	3.762	*(d)*	6.3	
*β‐Galactose (residue B)*					
B1	96.3	4.558	*(d)*	7.8	
B2	72.4	3.430	*(dd)*	7.8, 10.0	
B3	72.2	3.564	*(dd)*	3.3, 10.0	
B4	77.5	4.074	*(d)*	3.3	B4/C1
B5	75.0	3.713	*(dd)*	5.0, 7.7	
B6a	60.2	3.745	*(dd)*	5.0, 11.5	
B6b	60.2	3.777	*(dd)*	7.7, 11.5	
*Glucuronic acid (residue C)*					
C1a	102.6	4.379	*(d)*	8.0	C1a/A4
C1b	102.6	4.364	*(d)*	8.0	C1b/B4
C2	72.8	3.361	*(dd)*	8.0, 9.5	
C3	71.7 (or 75.2)	3.44–3.46		2nd order	
C4	75.2 (or 71.7)	3.44–3.46		2nd order	
C5	76.0	3.667		2nd order	
C6	175.5	—			

## 3 RESULTS

### 3.1 Analysis of six polysaccharide fractions from each of six members of the Viridiplantae

#### 3.1.1 Quantities of polysaccharide fractions

**FIG. 2:**
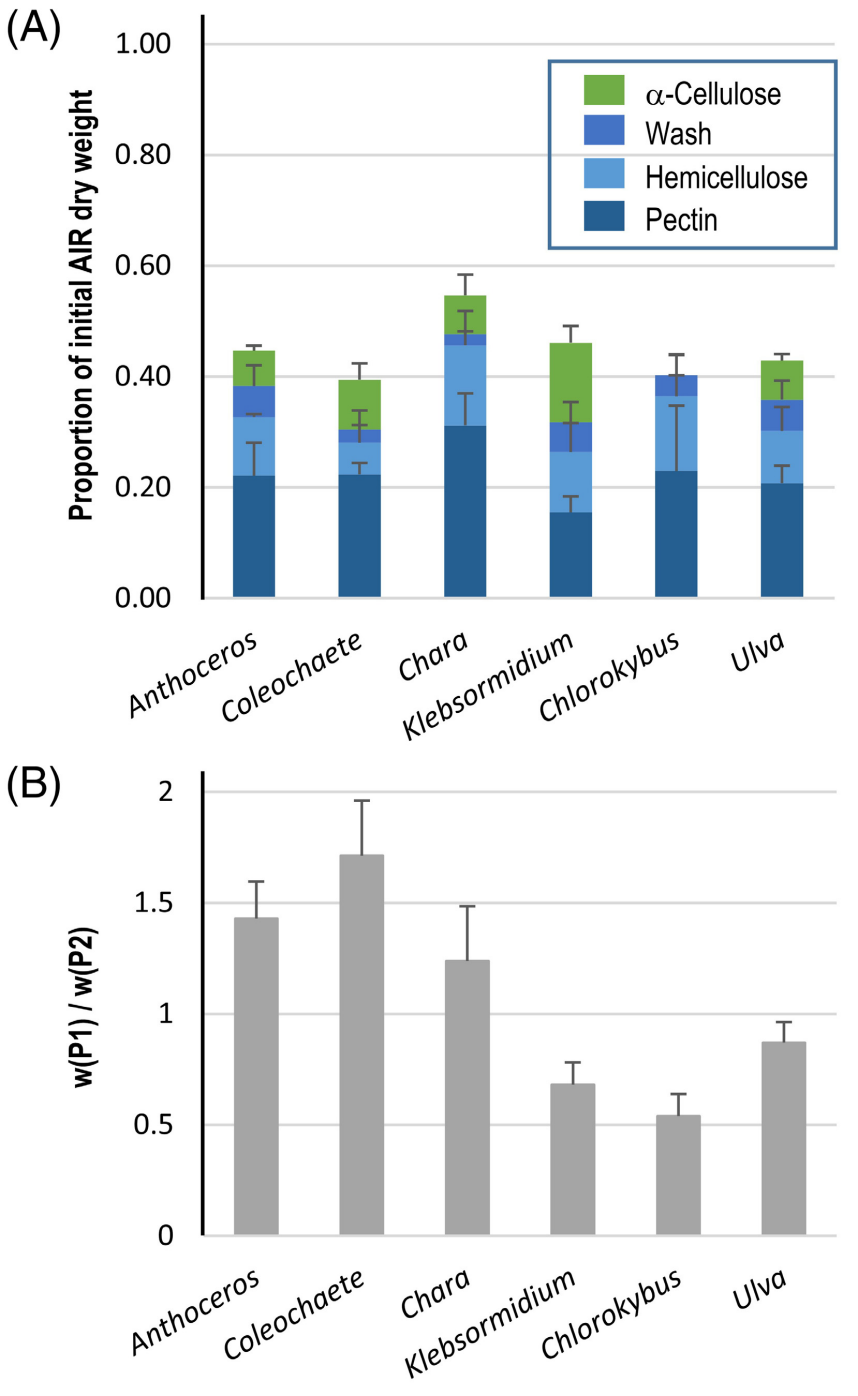
Ratio of polysaccharide fractions in six plant species. (A) Proportions of the different polysaccharide extracts, in various plants, relative to the initial AIR quantity. Owing to the small scale of the *Chlorokybus* sample, the Wash and α‐Cellulose fractions were not separated and are both presented as part of the Wash fraction. (B) Susceptibility of pectin to extraction (see text). The experiment was run in triplicate; error bars are SD, n = 3.

We first surveyed six species for cell wall composition: two early‐diverging charophytes (*Chlorokybus* and *Klebsormidium*), two later‐diverging charophytes (*Chara* and *Coleochaete*), and for comparison a chlorophyte (*Ulva*) and a non‐vascular embryophyte (*Anthoceros*). The AIR (total cellular polymers, rich in cell walls) was 60–80% of the cell dry weight. Polysaccharide classes were extracted sequentially (Fig. 2A). The total recovered yield was 40–55% of the AIR dry weight. ‘Pectin’ (extractable with hot oxalate) represented at least 30% of the total extracted mass. It was extracted in two steps: P1 after 2 h and P2 after a further 16 h. The susceptibility of pectin to extraction was evaluated by the ratio P1/P2, a high ratio indicating that much of the pectin was easily extracted (Fig. 2B). Extractability was higher in *Anthoceros* and in the later‐diverging charophytes than in the early‐diverging charophytes and *Ulva*; it was lowest in *Chlorokybus*, suggesting that *Chlorokybus* pectin has unusual properties. ‘Hemicellulose’ (extractable in concentrated alkali), and ‘α‐cellulose’ (the final insoluble residue) represented the other major parts of the AIR. Most of the extracted hemicellulose remained soluble after slight acidification (thus, it was ‘hemicellulose b’) but a minor proportion precipitated (giving ‘hemicellulose a’). The α‐cellulose proportion varied appreciably between the different plants, ranging from 5 to 45% of the total AIR; it was lowest in *Chlorokybus*. A further fraction, ‘Wash’, was obtained by extraction with a slightly acidic buffer from the alkali‐inextractable material and constituted a minor part of the total extracts. Overall, these results are comparable to previous findings (O'Rourke et al., 2015).

#### 3.1.2 Total sugar residue composition of polysaccharide fractions

**FIG. 3:**
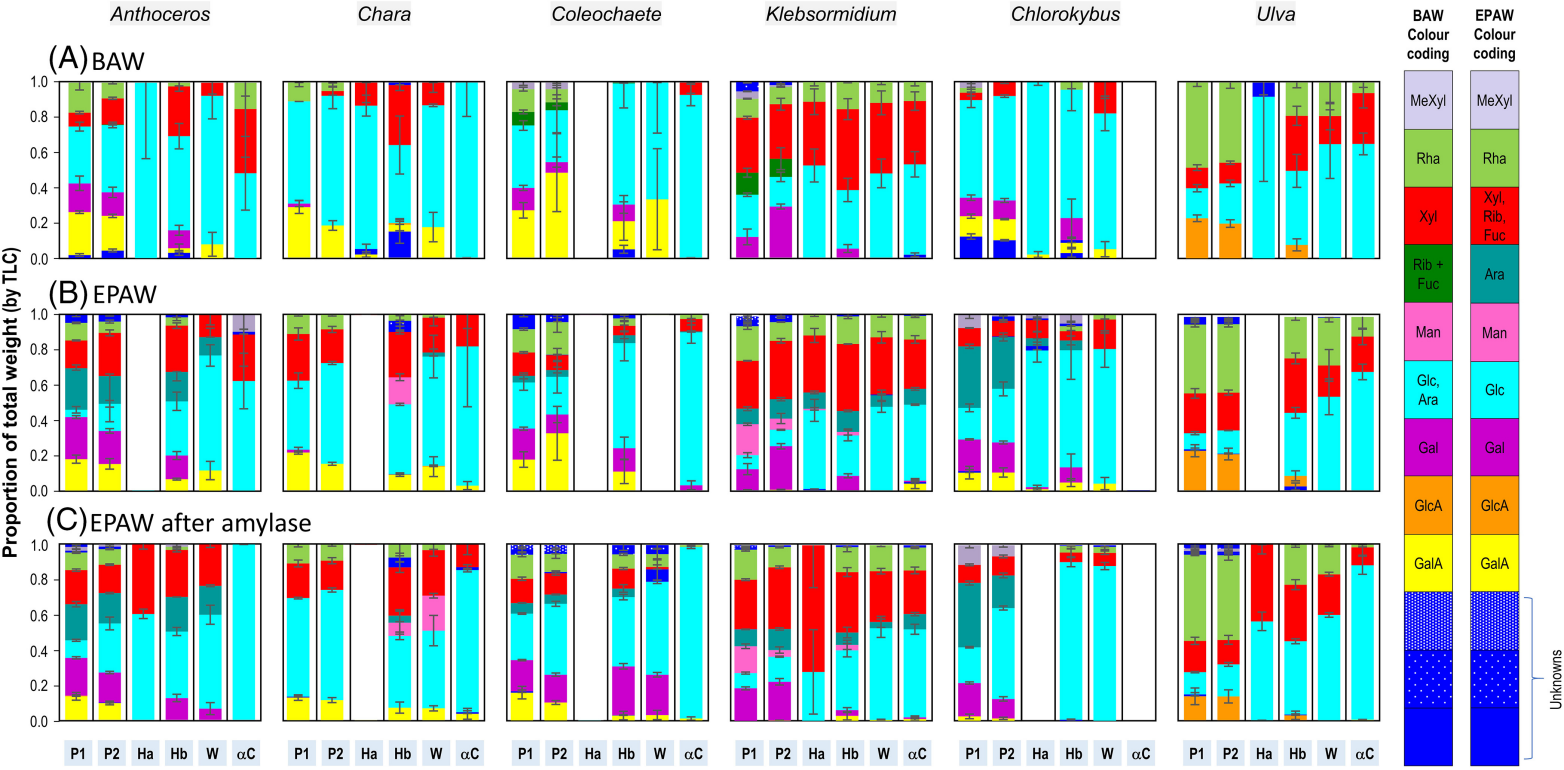
Relative quantities of sugar residues in six polysaccharide extracts from each of six plants. Quantification of hydrolysates prepared as in Fig. S1. The histograms show TFA hydrolysis products of polysaccharide fractions from (A,B) untreated AIR and (C) α‐amylase‐treated AIR. Chromatography solvents (always with two ascents) were: A, BAW; B,C, EPAW. Each TLC loading was derived from 15 μg of the polysaccharide fraction. Polymer fractions were: P1 and P2, ‘pectins’; Ha and Hb, hemicelluloses a and b; W, mildly acidic wash after alkaline extraction; αC, residual ‘α‐cellulose’. Sugars in the hydrolysates are colour‐coded. There are slight differences between the BAW and EPAW runs owing to differences in resolving power of the two solvent systems. Three aliquots of each AIR preparation were extracted to produce the listed polymer fractions, then three aliquots of each of the extracts were separately acid‐hydrolysed and analysed by TLC. Error bars are SD, with n = 3 (referring to the number of AIR preparations). ‘Unknown’ sugars could not be confidently identified with the markers.

The polysaccharide fractions were subjected to ‘complete’ acid hemicellulose and the products analysed by TLC in two solvent systems, revealing the total sugar residue composition, except cellulosic glucose (Figs 3A,B and S1). The chromatographic properties of all investigated sugars are listed in Table S2. BAW was more efficient for resolving uronic acids (Fig. S1A) and EPAW (Fig. S1B) for separating neutral sugars.

Many of the sugars detected were monosaccharides familiar from studies of land plants. In addition, however, at least 18 unfamiliar sugars were detected. *Chlorokybus* gave a relatively acid‐resistant, slow‐migrating aldobiouronic acid in its pectin and hemicellulose b fractions (Fig. S1A; labelled A1 and discussed further in §3.3). A known aldobiouronic acid (Popper et al. 2003) was confirmed to occur in *Anthoceros* (Fig. S1), and 3‐*O*‐methylgalactose (O'Rourke et al., 2015) was confirmed in *Chara* (Fig. S1B). Slow‐migrating unknowns were also detected in the hemicellulose b of *Chara*, *Coleochaete* and *Ulva*, and fast‐migrating unknowns were found in the pectins of all plants except *Chara* and in hemicelluloses of *Chlorokybus*, *Anthoceros* and *Ulva*. The colours of thymol staining give a preliminary indication of the nature of the unknowns (derivatives of hexoses, pentoses or deoxyhexoses; Table S2). Thus, algal polysaccharides exhibited appreciable differences from those of land plants, and it will be interesting in future studies to explore the ‘unknowns’.

In addition, a proportion of each AIR sample was de‐starched with α‐amylase. TLC showed that the amylase successfully released ethanol‐soluble oligosaccharides including maltose (Fig. S2). The AIR from all plants thus contained starch, as expected for the Viridiplantae (Busi et al., 2014; Sahoo and Seckbach, 2015; Necchi, 2016). The de‐starched AIR was also TFA hydrolysed and the products were analysed by TLC in EPAW — selected because it clearly resolves Glc, the acid hydrolysis product of starch, from all other common sugars (Fig. 3C).

Automated quantification of sugars on TLC plates was achieved (reported in Fig. 3) by use of an algorithm described in Rapin (2023). The analyses reported in Fig. 3 were conducted three times, allowing us to assess statistically the sugar compositions.

**FIG. 4:**
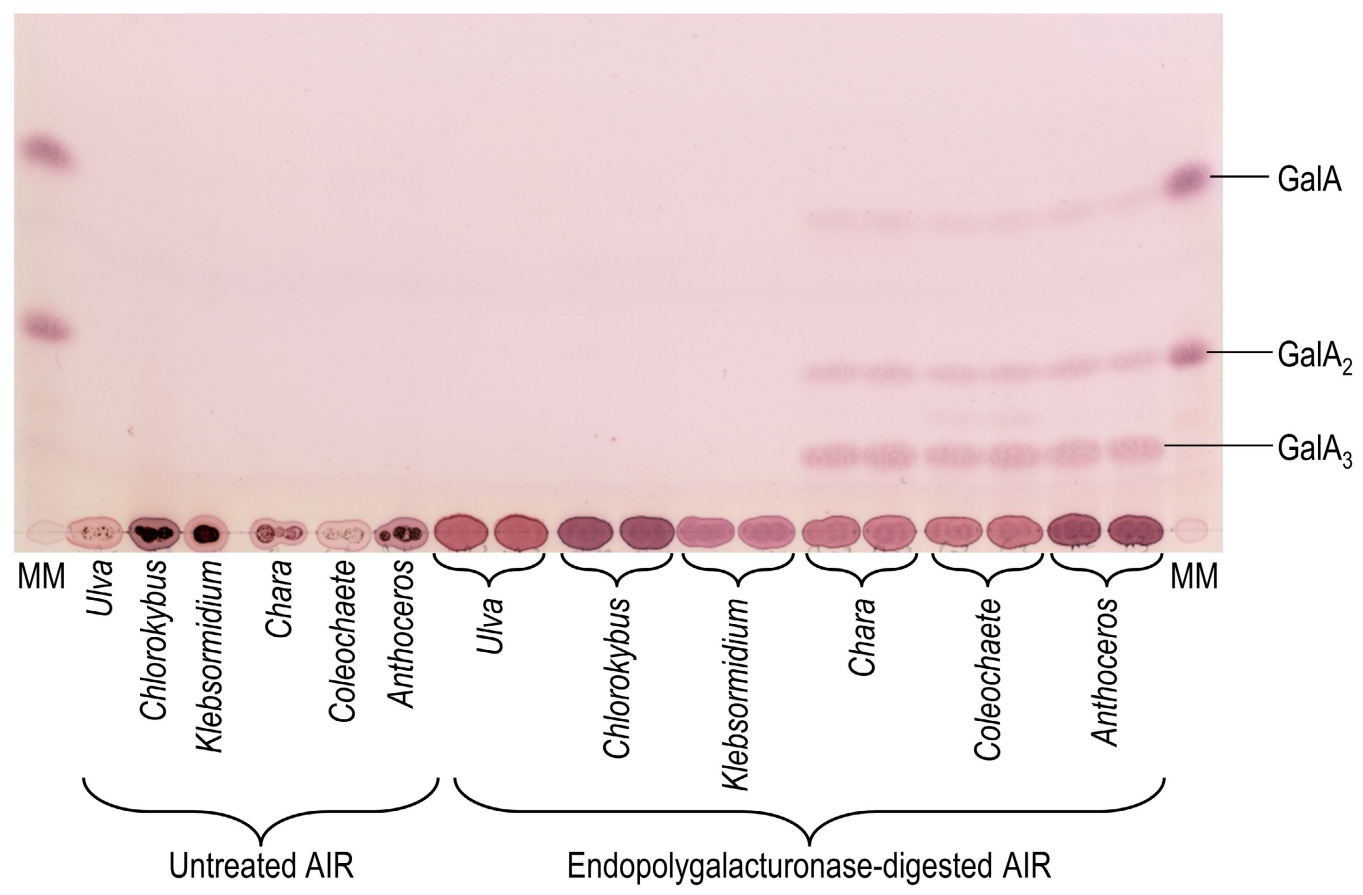
Endopolygalacturonase digestion of ‘pectic’ polysaccharides from six species of the Viridiplantae. AIR was de‐esterified with NaOH then digested with EPG, and the water‐soluble products were analysed by TLC on aluminium‐backed silica‐gel plates in BAW (2:1:1; single ascent). EPG alone gave no detectable spots. We loaded two replicate digests. Aqueous suspensions of the AIRs (without EPG treatment) were also loaded, demonstrating the absence of pre‐existing oligogalacturonides. MM, marker mixture of GalA and its disaccharide.

Concerning the uronic acids, which typically characterise pectins, Fig. 3 shows that all the streptophytes except *Klebsormidium* yielded GalA, especially in their pectic fractions, and *Chlorokybus* ‘pectin’ contained two uronic acids, probably GalA and GlcA. *Klebsormidium* gave no detectable uronic acids. The chlorophyte *Ulva* gave GlcA but no GalA. These results confirm and extend previous findings (Lahaye and Robic, 2007; Sørensen et al., 2011; O'Rourke et al., 2015). The AIRs of the six plant species were treated with EPG, which would be expected to hydrolyse homogalacturonan after de‐esterification in NaOH (Fig. 4). As expected, pectin from the land‐plant *Anthoceros* yielded the mono‐, di‐ and trisaccharide: GalA_1_, GalA_2_ and GalA_3_. The same result was obtained from the late‐diverging charophytes *Chara* and *Coleochaete*. However, the early‐diverging charophytes (*Chlorokybus* and *Klebsormidium*) and the chlorophyte (*Ulva*) gave no oligogalacturonides and thus do not contain homogalacturonan.

The main neutral sugar residues in ‘pectins’ (Figs 3 and S1) were usually Gal (*Anthoceros*, *Coleochaete*, *Klebsormidium*, *Chlorokybus*), Ara (in *Anthoceros*, *Klebsormidium*, *Chlorokybus* and to some extent *Coleochaete*), Rha (in all the plants except *Chlorokybus*), and variable amounts of Glc. Xyl was present abundantly in the ‘pectins’ of *Klebsormidium* and *Ulva* and in small amounts in those of *Anthoceros* and *Chlorokybus*; and *Chara* and *Coleochaete* gave almost none. Uniquely, *Klebsormidium* ‘pectin’ was rich in Man, which, however, was difficult to quantify on the BAW chromatogram.

Fig. S1 shows that hemicellulose a (the portion of the alkaline extract that precipitates upon slight acidification) was a very minor component of most of the plants (*Anthoceros*, *Chara*, *Coleochaete* and *Ulva*), but present in the early‐diverging charophytes — *Klebsormidium* (principally Glc and Xyl residues) and *Chlorokybus* (largely composed of Glc residues). Hemicellulose b (remaining soluble upon acidification) was always the more abundant of the two hemicellulose fractions; it was always rich in Glc residues and often also in Xyl (*Anthoceros*, *Chara*, *Klebsormidium* and *Ulva*, but not *Coleochaete* and *Chlorokybus*). Hemicellulose b was relatively Man‐rich in *Chara*, but not in the other five species.

Sugar residues were consistently present in the wash fraction (W; Figs 3 and S1), as has also been reported in many land‐plant studies (de Castro et al., 2017), albeit without a satisfactory explanation. In our work, fraction W was always rich in Glc residues and to a lesser extent Xyl. The W fractions extracted from untreated AIR were only slightly richer in Glc than those from amylase‐digested AIR, showing that most amylase‐resistant starch had been extracted during previous steps. The α‐cellulose fraction (remaining insoluble after all extractions and expected to be relatively resistant to TFA hydrolysis owing to the crystallinity of cellulose) also gave Glc and smaller amounts of Xyl. The low yield of αC obtained from *Chlorokybus* did not give quantifiable sugars on TFA hydrolysis.

### 3.2 Unique features of *Chlorokybus* ‘pectin’

It is clear from the above results that the six species tested have several polysaccharide features in common but perhaps a larger number of important differences, even within the charophytes. Pectins were particularly diverse — which was predictable in the chlorophyte (*Ulva*) but perhaps less expected within the streptophytes. This prompted us to examine in more detail the pectin from *Chlorokybus* (the earliest‐diverging streptophyte tested), both as a step towards understanding the evolutionary origin of land plant pectin, and as an indication of the adaptation of extant *Chlorokybus* to its habitat.

**FIG. 5:**
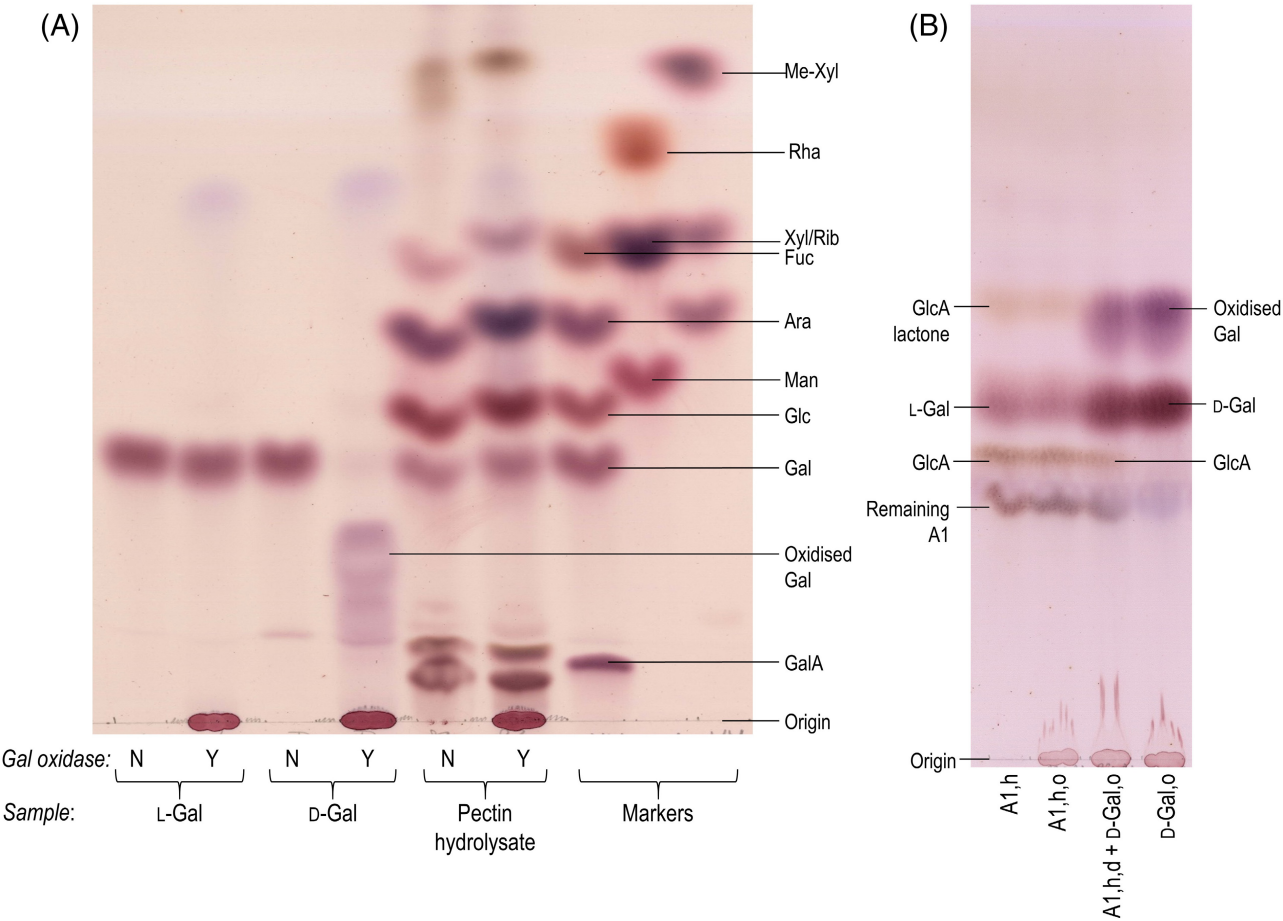
The galactose in *Chlorokybus* pectin and in its aldobiouronic acid (A1) are the l‐enantiomer. (A) Whole *Chlorokybus* pectin P2 was acid‐hydrolysed then treated (Y), or not (N, incubated in enzyme buffer), with d‐galactose oxidase. Products were analysed by TLC (in EPAW). For comparison, commercial l‐ and d‐galactose were treated, or not, with d‐galactose oxidase. (B) Purified A1 (see Fig. 6A) was re‐hydrolysed with TFA (A1,h), then treated with d‐galactose oxidase (A1,h,o), and mixed with galactose oxidase‐treated d‐Gal (A1,h,o + d‐Gal,o). A sample of oxidase‐treated d‐Gal (d‐Gal,o) was run for comparison. The TLC was developed in BAW with one ascent. Abbreviations: h, hydrolysed in TFA; o, incubated with galactose oxidase.

*Chlorokybus* pectin displayed up to 25% of uronic acid residues (Fig. 3). However, these were hard to qualify properly. The *Chlorokybus* compound labelled ‘Unknown’ in Fig. 3 migrated much slower than uronic acid monomers. It is probably an aldobiouronic acid, possibly resembling the α‐d‐glucuronosyl‐(1→3)‐l‐galactose known from *Anthoceros* polysaccharides (Popper et al., 2003), and is further investigated in §3.3. GalA and GlcA were not always clearly distinct nor did they always exactly match the markers, probably because of distortion caused by contaminating non‐sugar products in the hydrolysates. Three neutral sugars constituted the bulk of *Chlorokybus* de‐starched pectin: galactose, glucose and arabinose, which made up respectively ~20, 20 and 40% of the sugar residues in P1, and ~10, 35, and 25% in P2. Interestingly, the amount of glucose did not decrease after α‐amylase digestion, indicating that it was not principally due to contaminating starch. The enantiomerism of the Gal was tested by incubation with commercial d‐galactose oxidase, which we confirmed does not act on l‐Gal (Fig. 5A). Most, if not all, of the Gal in the *Chlorokybus* pectin hydrolysate was unaffected by the oxidase (Fig. 5A), indicating that it was l‐Gal, which is generally the rarer enantiomer in nature. Xylose was a minor compound of *Chlorokybus* pectin. A fast‐migrating compound, staining brownish like galactose, was visible in *Chlorokybus* pectin in the EPAW solvent system.

### 3.3 Characterisation of potentially novel components of *Chlorokybus* pectin

**FIG. 6:**
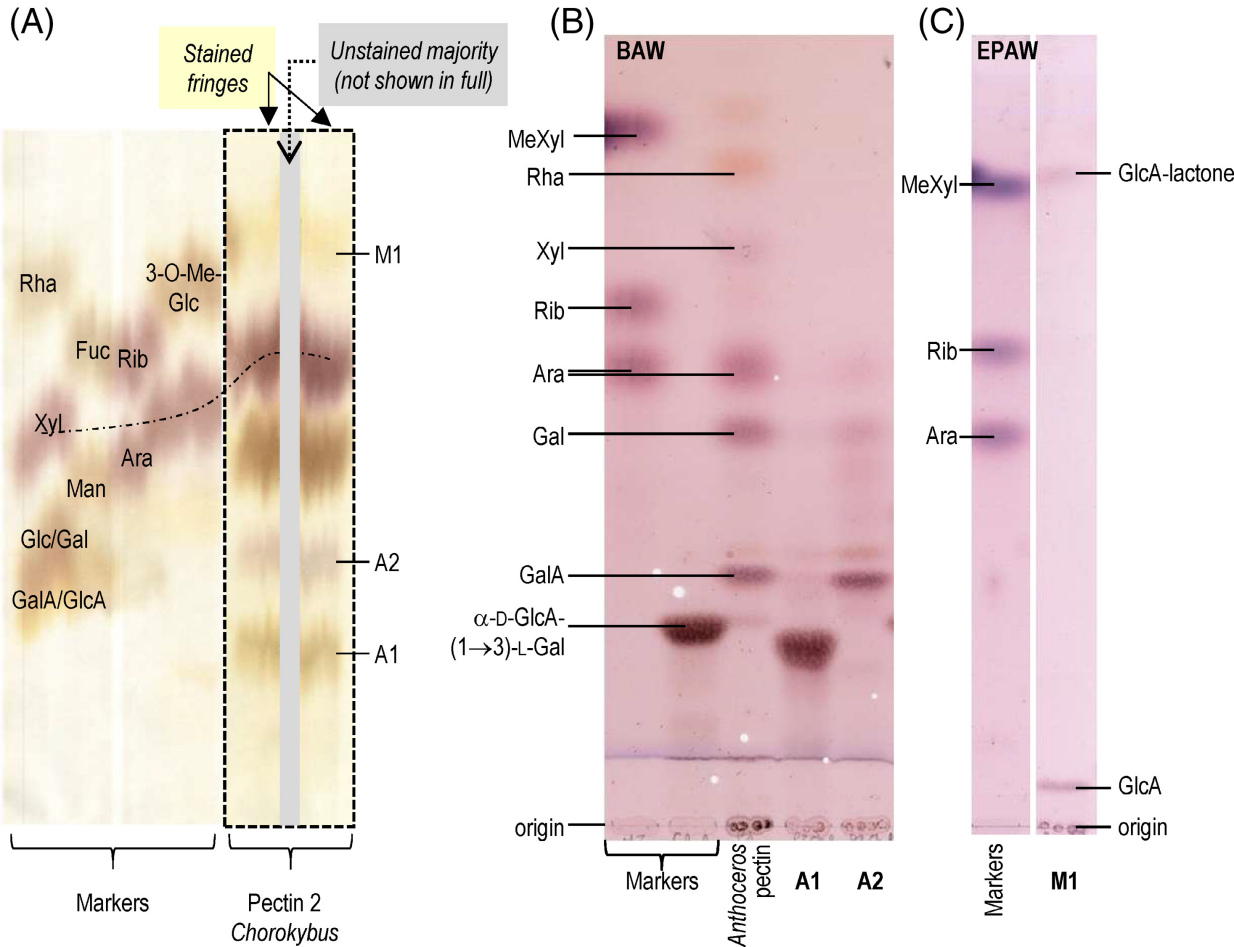
Chromatography of acid hydrolysate of pectin 2 from *Chlorokybus*. The pectin was hydrolysed with 2 M TFA for 1 h at 120°C. (A) Preparative paper chromatography of the total hydrolysate. Chromatography solvent BAW (12:3:5), run for 24 h. The fringes of the preparative loading were stained with aniline hydrogen‐phthalate, then the unstained portion of interesting compounds (not shown here in full), A1, A2 and M1, were eluted for further analysis. The dashed black line indicates the position of Xyl on the paper, as a visual guide. (B,C) TLC of small portions of A1, A2 and M1 from (A). Markers included an *Anthoceros* pectin hydrolysate containing the aldobiouronic acid α‐d‐GlcA‐(1→3)‐l‐Gal (Popper et al., 2003). The solvents were (B) BAW (4:1:1) and (C) EPAW (6:3:1:1).

Some compounds in the *Chlorokybus* pectic hydrolysate could not initially be identified, as they did not correspond to the most common sugar residues used as markers (Fig. S1). We explored these components in more detail in the pectin 2 (P2) polysaccharide fraction. The TFA hydrolysate of *Chlorokybus* P2 was subjected to preparative paper chromatography (PPC; Fig. 6A). Part of the paper was stained with aniline hydrogen‐phthalate, revealing reducing sugars. The colour of the staining depends on the nature of the reducing terminus: uronic acids, orange; neutral hexoses, brown; neutral pentoses, red. Three unknowns were selected: A1 (‘anion’, slow‐migrating), M1 (‘methyl and/or deoxy’, fast‐migrating compounds), and A2 (second ‘slow‐migrating’ anion). A1, M1 and A2 were eluted from the unstained part of the PPC and small proportions run on TLC (Fig. 6B,C).

M1 migrated in the vicinity of the relatively hydrophobic sugars (methylated and/or deoxy monosaccharides) on PPC, but stained orange with aniline hydrogen‐phthalate, suggesting a uronic acid‐reducing terminus (Fig. 6A). On TLC, it displayed two bands, both staining purple with thymol: one migrating (slowly) in the uronic acid zone, the other (fast) in the ‘methyl or deoxy’ sugar zone (Fig. 6C). They thus appeared to be the open‐chain and lactonised forms, respectively, of a single uronic acid that had been interconverted after elution from the PPC. The anion ran in the vicinity of GlcA or GalA. However, GalA does not lactonise; therefore, M1 in Fig. 6A is proposed to be GlcA lactone.

A2 migrated with anionic uronic acids on PPC, but stained reddish with aniline hydrogen‐phthalate, suggesting a pentose at the reducing terminus (Fig. 6A). However, on TLC in BAW, A2 appeared to be mainly a mixture of several known sugars, including GalA, Gal, and Ara and was not investigated further (Fig. 6B).

A1 stained brown with aniline hydrogen‐phthalate, suggesting a neutral hexose at the reducing terminus, and it ran considerably slower than GalA on PPC (Fig. 6A). On TLC (Fig. 6B) it migrated slightly slower than the known aldobiouronic acid α‐d‐GlcA‐(1→3)‐l‐Gal (which is relatively resistant to acid hydrolysis and was previously characterised from *Anthoceros* [Popper et al., 2003]). When A1 was re‐hydrolysed with TFA, it yielded GlcA and Gal (Fig. 5B, left‐hand lane). A1 thus seemed very similar, but not identical (Fig. 6B), to α‐d‐GlcA‐(1→3)‐l‐Gal. They could differ by linkage, anomerism and/or the sugar enantiomer(s) involved.

The optical isomerism of the galactose was tested. d‐Gal and l‐Gal co‐migrate on TLC so, in order to distinguish them from one another, we incubated the hydrolysed A1 with d‐galactose oxidase, which selectively oxidises d‐ but not l‐Gal (Fig. 5A and Popper et al., 2003). This indicated that the Gal residue present in A1 was l‐Gal, the less common of the two enantiomers (Fig. 5B). The GlcA residue is assumed to be d‐ and not l‐ since l‐GlcA is unknown in plants. Thus A1 is proposed to be an d‐GlcA‐(1→?)‐l‐Gal. This disaccharide was further characterised by NMR spectroscopy.

The proton NMR spectra (selective 1‐D TOCSY spectra and 2‐D COSY) of A1 confirm the presence of α‐ and β‐l‐Gal anomers. The COSY cross‐peaks identify protons that are up to three chemical bonds apart and these, together with the reciprocal coupling constants, allow assignation of chemical shifts to all protons in A1 (Fig. S3; Table 1). The presence of a (1→4) linkage was suggested by crosspeaks in the NOESY spectrum (Fig. S4; nuclear Overhauser effect spectroscopy, which shows the interaction of protons close in space) between the H‐1 proton of GlcA and the H‐4 protons of the reducing Gal (both α and β). The (1→4) linkage was confirmed by the HMBC spectrum (heteronuclear multiple bond correlation; showing correlations between carbon and proton nuclei separated by 2 or 3 bonds; not included in the figures), which showed correlations between C‐1 of the GlcA residue and the Gal H‐4 proton, and between Gal C‐4 and the H‐1 of GlcA.

The large coupling constant (8.0–9.5 Hz; Table 1) between H‐1 and H‐2 of GlcA showed H‐1 to be in an axial position, indicating the glycosidic linkage is β. It is not possible to determine from NMR data alone whether the individual residues are d‐ or l‐; this was determined in the case of Gal by use of d‐galactose oxidase. This was supported by the NOESY spectrum which showed through‐space crosspeaks between GalA H‐1 and GalA H‐3 and GalA H‐5, consistent with an axial (β) GalA H‐1, rather than an equatorial (α) one.

In conclusion, A1 is the novel disaccharide β‐d‐Glc*p*A‐(1→4)‐l‐Gal (Fig. S4), thus differing from the *Anthoceros* dimer in anomeric configuration of the d‐GlcA (α) and the locant (1→3). We were not able to find any previous reports of the A1 structure.

### 3.4 Characterisation of the *Chlorokybus* ‘pectic’ polysaccharide(s)

#### 3.4.1 Enzymic digestion of *Chlorokybus* ‘pectin’

**FIG. 7:**
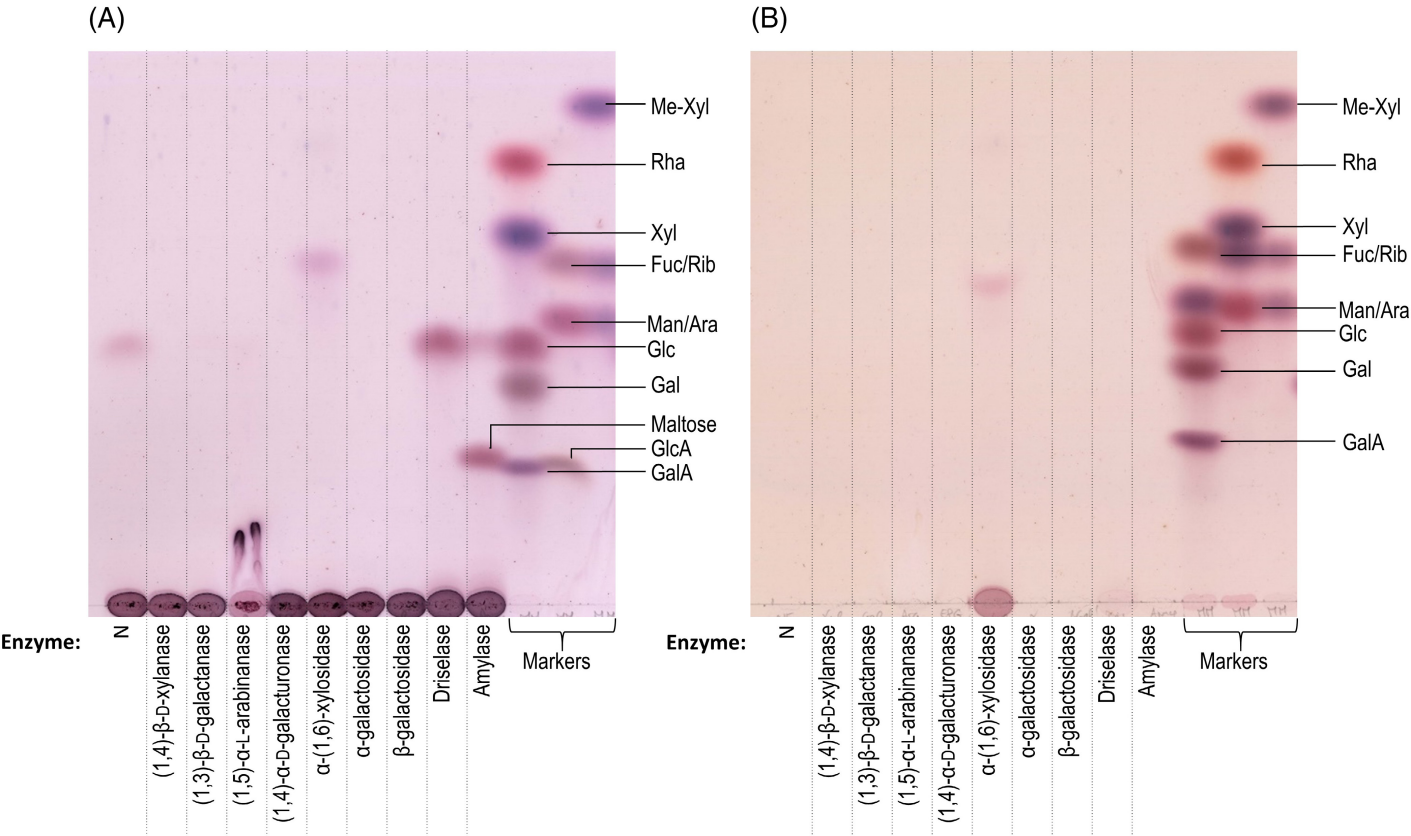
Enzymic digests of *Chlorokybus* ‘pectin’. (A) ‘Pectin’ fraction P2 from *Chlorokybus* was treated with a series of endo‐ and exo‐hydrolases. N = no enzyme (pectin incubated alone for 72 h). (B) Substrate‐less controls of the same enzyme collection. Incubation conditions were as in Table S1. The TLC plates in were developed twice in BAW.

In order to further characterise the intriguing *Chlorokybus* ‘pectin’, we applied a series of endo‐ and exo‐hydrolases to intact *Chlorokybus* polysaccharide fraction P2 (Fig. 7): (1,5)‐α‐l‐arabinanase, (1,4)‐β‐d‐galactanase, EPG, and α‐ and β‐galactosidases. In land plant pectins, arabinanase and galactanase are expected to hydrolyse neutral pectic sidechains, whilst EPG digests homogalacturonan (Bonnin et al., 2002). All enzymes yielded negative results (including EPG, agreeing with Fig. 4): thus, if *Chlorokybus* P2 contains any classical land‐plant pectic structures, the chosen enzymes did not hydrolyse them (Fig. 7).

Hemicellulose‐digesting enzymes were also used: (1,4)‐β‐d‐xylanase and α‐(1,6)‐xylosidase (respectively aimed at xylan and xyloglucan structures). They had no detectable effect on P2 of *Chlorokybus* (Fig. 7). An unidentified band was visible in the α‐xylosidase track; however, it was also present in the substrate‐less control and is assumed to be a contaminant of this enzyme.

Finally, amylase (aimed at starch) and Driselase were tried. Driselase is able to digest all cell wall polysaccharides of land plants except rhamnogalacturonan‐II (Fry, 2010). Both yielded only glucose, plus maltose in the case of amylase, which were not visible in the substrate‐less controls (Fig. 7). Thus, a certain amount of starch was evidently present in the pectic extract. A trace of glucose was also observable in the enzyme‐less control: this reaction mixture had been incubated for the longest incubation time (coinciding with those of amylase and Driselase) at the highest temperature (37°C). These conditions might have provoked the degradation of a glucose‐containing polymer such as starch (Pigłowska et al., 2020). Driselase is unlikely to contain l‐galactosidase activity, although it does contain activities that might have been expected to release other monosaccharides present in *Chlorokybus* pectin: Ara (assumed to be l‐), and Xyl and GalA (both assumed to be d‐). Driselase contains β‐d‐xylosidase (but not α‐d‐xylosidase), α‐l‐arabinosidase (but not β‐l‐arabinosidase), and α‐d‐galacturonidase. Thus, the Ara, Xyl and GalA residues of *Chlorokybus* pectin are unlikely to be α‐l‐Ara, β‐d‐Xyl and α‐d‐GalA.

#### 3.4.2 Fractionation of *Chlorokybus* ‘pectin’ on anion‐exchange chromatography

To investigate the heterogeneity of *Chlorokybus* ‘pectin’ P2, we fractionated it on Q‐Sepharose, a strong anion‐exchange resin, as described by Popper and Fry (2005).

**FIG. 8:**
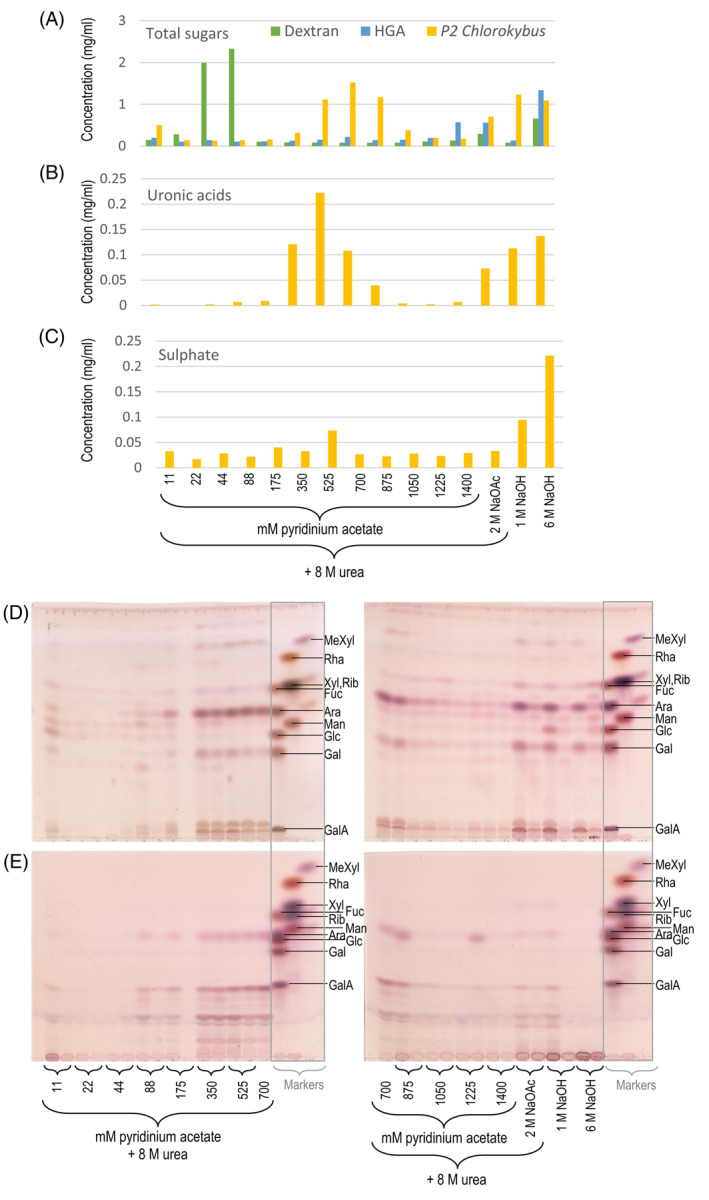
Fractionation of intact *Chlorokybus* P2 ‘pectin’ on anion‐exchange chromatography. Prior to analyses, the urea was removed by dialysis. (A) Profile of total sugars, measured by a thymol dot‐blot assay. Dextran and homogalacturonan (‘HGA’) were run on the column as respectively neutral and anionic standards. (B) Profile of uronic acid residues, measured by *m*‐hydroxybiphenyl assay. (C) Sulphate profile, measured by a barium acetate assay complete acid hydrolysis of the fractions. (D) TLC of fully hydrolysed fractions from *Chlorokybus* P2 (2 M TFA, 120°C, 1 h). (E) TLC of mildly hydrolysed fractions (0.1 M TFA, 100°C, 2 h). For (D) and (E), each fraction comprised two samples collected successively in each indicated eluent. TLC plates were developed in EPAW for (D), and in BAW for (E). The marker mixtures (in grey rectangles) interrupt the sequence of fractions.

Neutral dextran and highly anionic homogalacturonan were applied to the column for calibration (Fig. 8A). As expected, dextran was scarcely retained on the column, being largely eluted with 44–88 mM pyridinium acetate. In contrast, homogalacturonan interacted strongly with the beads and was only released by the last eluents (1400 mM pyridinium acetate, 2 M sodium acetate and 6 M NaOH). On the same column, *Chlorokybus* pectin P2 exhibited two main peaks, eluting at ~350–875 mM buffer and ~1 M NaOH, respectively. The earlier peak accounted for the majority of the uronic acid residues (Fig. 8B). The later peak was richer in sulphate groups, which presumably accounted for its highly anionic nature (Fig. 8C). Sulphate was not a previously known component of streptophyte pectins, though sulphated peptides are known in land plants (De Giorgi et al. 2021) and sulphated pectin‐like polymers have long been known in marine algae (Percival, 1979).

The ‘pectin’ fractions were analysed for sugar residue composition after ‘complete’ acid hydrolysis (Fig. 8D). The earlier‐eluting pectin was rich in Ara, Gal and uronic acids (both GalA and GlcA), and contained Xyl as a minor component. The later‐eluting pectin was surprisingly similar to the earlier‐eluting pectin in terms of sugar composition, apart from a Glc band in the 1 M NaOH fraction. For each of the later eluents, sugars were predominantly detectable in the first of the two collected portions, showing that little additional polysaccharide would have been eluted if these eluents had been continued. There was a correlation between the quantity of GlcA and neutral Gal in each fraction, supporting the idea that most of the GlcA and Gal were linked, presumably in the form of the novel aldobiouronic acid, β‐d‐GlcA‐(1→4)‐l‐Gal.

Other aliquots of the ion‐exchange fractions were submitted to mild acid hydrolysis. From the first peak of pectin, monomeric Ara was released, suggesting that it was in the furanose ring‐form. An unidentified band migrated slightly slower than the GalA marker and stained a slightly different colour from authentic GalA; it may have been a disaccharide such as Gal‐Ara that had been linked within the polysaccharide as Gal*p*‐Ara*f* (where *f* and *p* indicate the furanose and pyranose ring‐forms), of which only the Ara*f* residues were appreciably susceptible to hydrolysis by mild acid (Albersheim et al., 2011). In addition, the previously characterised β‐d‐GlcA‐(1→4)‐l‐Gal was generated as well as several lower‐mobility oligomers. The efficient release of the disaccharide β‐d‐GlcA‐(1→4)‐l‐Gal by mild acid raises the possibility that its l‐Gal moiety had been present in the polysaccharide as l‐Gal*f*. Samples from the second peak, however, were only marginally hydrolysed: they released the same compounds in much smaller quantities, and much polysaccharide material remained immobile at the origin of the TLC, especially for the 6 M NaOH eluate.

Overall, separation on anion‐exchange chromatography allowed for the release of two distinct fractions with similar sugar compositions, one being richer in uronic acids and susceptible to mild acid hydrolysis, the other one being richer in sulphate groups (hence the higher negative charge) which remain at least partially attached during mild acid hydrolysis and containing less‐labile glycosidic bonds.

## 4 DISCUSSION

In land plants, the cell wall is seen as a conjugate of three main polymer fractions with clearly distinct roles: pectin (an acidic complex of linear and branched polysaccharide domains forming a gel via ionic interactions with calcium cations, borate esters and probably hemicellulose–pectin glycosidic bonds), hemicelluloses (a largely neutral network of linear polysaccharides that hydrogen‐bond strongly with cellulose), and cellulose (a rigid, straight‐chain polysaccharide that forms crystalline microfibrils providing mechanical strength to the cell walls). However, the key features of the object of this study, *Chlorokybus atmophyticus*, hint strongly at very different biological, botanical, physical and chemical properties. Indeed, *Chlorokybus*, as a 2‐ to 4‐cell sarcinoid organism living in moist environments, may not need a wall structure as mechanically strong as those in a larger multicellular plant; on the contrary, *Chlorokybus* pectin may help the cells to adhere to their surface substrata and/or to retain water.

The present paper focuses on pectin, particularly in *Chlorokybus*. In land plants, pectin usually comprises three main domains: homogalacturonan, rhamnogalacturonan‐I and rhamnogalacturonan‐II. Each of these domains fulfils a variety of roles within the cell wall at different developmental stages of the organism. More precisely, homogalacturonan, thanks to its gelling properties, is known for being key in cell–cell interactions, regulation of cell wall pores and permeability, and cell–substratum adhesion (Domozych and Domozych, 2014; Domozych et al., 2014a). Rhamnogalacturonan‐I (RG‐I) (consisting of a backbone of alternating GalA and Rha, with Gal‐ and/or Ara‐rich sidechains) plays a role in maintaining the structural integrity of the plant, managing mechanical changes in the cell wall during fruit ripening, playing a role in cell–cell adhesion, managing water deficits and stomatal opening (in conjunction with homogalacturonan), through changes in its fine structure and in its interactions with other cell wall polysaccharides (Zykwinska et al. 2007, 2008; Kaczmarska et al., 2022). RG‐I is ubiquitous amongst land plants; however, its finer structure varies considerably depending on the species and developmental stage (Albersheim et al., 2011). Finally, rhamnogalacturonan‐II (RG‐II), which is the most complex known polysaccharide (made of a GalA backbone and substituted with eleven different glycosidic residues organized in multiple different sequences), with its ability to form borate diester bridges, is thought to play a role in plant morphology and mechanical properties (O'Neill et al., 2004; Séveno et al., 2009; Begum & Fry, 2022). The structure of RG‐II is highly conserved amongst land plants, suggesting a fine‐tuning of its properties in the cell wall (Caffall and Mohnen, 2009).

Of these three polymeric structures, only one, homogalacturonan, has been chemically detected repeatedly in the charophytes (Cherno et al., 1976; Popper et al., 2011; O'Rourke et al., 2015). Even though the sugars present in the cell walls of later‐diverging charophytes suggest the presence of RG‐I, and RG‐I‐like epitopes have been detected immunologically in the charophyte *Penium* (Domozych et al., 2014b), RG‐I has not been chemically proven in charophytes (O'Rourke et al., 2015). Wang et al. (2020) have reported the presence in *Chlorokybus* of a gene that encodes a ‘GALS' galactosyltransferase potentially contributing to RG‐I synthesis; however, this protein's enzyme activity has apparently not been tested. Such annotations cannot be considered proof of any postulated role in polysaccharide biosynthesis, which must necessarily be anchored in reliable polysaccharide analysis. Likewise, *Chlorokybus* has four genes encoding putative ‘xyloglucanase’ GH77 proteins and two encoding ‘α‐xylosidase’ GH31 proteins (Wang et al., 2020), although it lacks the proposed substrate — xyloglucan. RG‐II has also not been detected in charophytes, to the best of our knowledge.

The first key finding of the present study is the extractability of pectin (with hot ammonium oxalate): it is much lower in early‐diverging charophytes than in *Chara*, *Coleochaete* and the bryophyte *Anthoceros*. This difference in extractability also correlates with the cell wall susceptibility to EPG, and therefore the presence of homogalacturonan: susceptibility to EPG seems to pair up with higher pectin extractability. As the ammonium oxalate extraction protocol was designed for land plants (rich in homogalacturonan) and is based upon the breaking of ionic bonds (calcium cation–carboxylic acid), it is not surprising that it is more efficient on more land‐plant‐like cell walls such as those of *Chara* and *Coleochaete*. This indicates that the pectic fraction of uronic acid‐containing cell walls from early‐diverging charophytes, i.e. *Chlorokybus*, is not based around the same cation–carboxylic acid gel. However, homogalacturonan‐based interactions in the cell walls underlie many plant functions, such as cell–cell interactions, regulation of cell wall pores and permeability, and cell–substratum adhesion (Domozych and Domozych, 2014; Domozych et al., 2014a). *Chlorokybus* is a small semi‐terrestrial alga, lacking a cuticle but able to endure periods of drought, so it must have developed alternative mechanisms for delivering such functions via widely different molecular mechanisms.

Presence of another key land‐plant polysaccharide, rhamnogalacturonan‐I (RG‐I), is also lacking chemical evidence throughout the charophytes, including *Chlorokybus*. Rhamnose, which makes up half of the backbone of RG‐I (repeats of [→4)‐α‐d‐GalA‐(1→2)‐α‐l‐Rha‐(1] dimers), is essentially absent from *Chlorokybus* ‘pectin’ (Figs 3 and S1). However, a novel dimer extracted from *Chlorokybus* ‘pectin’ was characterised, which conjugates a uronic acid and a neutral sugar: β‐d‐GlcA‐(1→4)‐l‐Gal. This aldobiouronic acid is unique in many aspects. Firstly, it features glucuronic acid, which is usually found as a sidechain in hemicelluloses or in RG‐II (Scheller and Ulvskov, 2010; Kaczmarska et al., 2022). UDP‐GlcA being an intermediate for the biosynthesis of UDP‐GalA (Caffall and Mohnen, 2009), it could be that the bio‐machinery leading to GalA synthesis is modified in *Chlorokybus*, leading to a greater GlcA incorporation during cell‐wall polysaccharide synthesis. Secondly, this novel dimer features l‐Gal, which is the less common Gal enantiomer found in plants. This enantiomer has only been detected as traces in green plants, occurring in RG‐II (Pabst et al., 2013) and replacing l‐Fuc in the xyloglucan of certain plants (e.g. jojoba [Hantus et al., 1997]), as well as occurring as part of an aldobiouronic acid in the bryophyte *Anthoceros caucasicus* (Popper et al., 2003). None of these three instances seem to be related to the structure at hand, as they are evolutionarily particularly distant from *Chlorokybus*. Moreover, upon chemical dissection of the pectic fractions from *Chlorokybus*, it was found to contain l‐Gal in abundance, and little or no d‐Gal. This peculiarity could explain the resistance of the *Chlorokybus* cell wall to the classic cell wall enzymes β‐galactanase and α‐ and β‐galactosidases. Moreover, during ‘pectin’ acid hydrolysis, l‐Gal was released at the same rate as GlcA, indicating their close association.

Arabinose, another neutral monomer usually abundant in land‐plant cell walls, was also investigated. It also was not released by the classic cell wall enzyme α‐l‐arabinanase, or by the α‐l‐arabinosidase activity of Driselase, although it was readily released during mild acid hydrolysis, indicating that it was present in its usual furanose form (as in land‐plant pectins). One possibility is that the Ara is present as β‐l‐Ara*f* residues, as found in the evolutionarily ancient hydroxyproline triarabinoside core of extensin‐like glycoproteins (Bollig et al., 2007). Taken together, these unique *Chlorokybus* features could point towards novel pectic chemical structures, with the dimer β‐d‐GlcA‐(1→4)‐l‐Gal forming a semi‐acidic backbone substituted by Ara units, linked together via as‐yet unknown glycosidic bonds. Such a structure would be similar to RG‐I (with a backbone of GalA‐Rha, and galactan and arabinan sidechains). Such an analogous polymer could therefore be playing similar roles, interacting with the fibrous cellulosic backbone to guarantee the alga's mechanical integrity (Zykwinska et al., 2008) or protecting it from desiccation (Moore et al., 2008).

However, considering the pectic structure of *Chlorokybus* as homogeneous would be to overlook some major findings of our work. Indeed, *Chlorokybus* pectin is clearly made of two fractions, the first one being less negatively charged, more susceptible to mild acid hydrolysis, richer in uronic acids and poorer in sulphates than the second one. The presence of sulphates within the charophytes seemed counter‐intuitive, as cell wall polysaccharide sulphation has often been thought of as a feature of adaptation to saline environments (Aquino et al., 2011). It may be a trait ancestral to chlorophytes and streptophytes, which was eliminated after the divergence of the Chlorokybophyceae. It might also be that *Chlorokybus* cells, being embedded in mucilage in close contact with mineral‐rich environments (rock, soil), effectively also occupy highly saline environments (as mineral ions from the rock might partially dissolve in the mucilaginous matrix) thus provoking the need for similar adaptations to those seen in a highly saline marine environment (Tosif et al., 2021).

Taking these observations together, we propose that an RG‐I‐like structure may be present within *Chlorokybus* cell walls. On the one hand, the non‐sulphate‐containing, more land‐plant‐like fraction could still be responsible for the previously cited roles of maintaining the alga's mechanical integrity and interacting with other cell wall polymers. On the other hand, the almost similar, sulphate‐containing, more negatively charged fraction could play the role of regulating exchanges with its natural mineral‐rich environment and potentially form a gel via as‐yet undescribed ionic interactions with potentially present cations. Confirming the existence of such a 'pectic' organisation would require a full characterisation of the linkages within the cell wall, potentially via further enzymic screening of the polysaccharides and the use of spectrometric techniques to analyse bigger polysaccharide fragments. Studying the crystalline structure and molecular interactions of this polymer would shed light on its potential structural similarity with RG‐I or with other known polysaccharides. Moreover, studying the potential dynamic sulphation features of *Chlorokybus* would help with understanding the role of sulphation in a non‐marine alga. Studying the biosynthetic machinery — and the genes involved in it — would shed light on the origins of this unique polymeric structure and help in understanding whether it derives from pre‐existing features that were later erased, or whether *Chlorokybus* evolved independently after diverging from all other charophytes.

## 5 CONCLUSIONS

In conclusion, we describe in this paper the structure of the hot‐oxalate‐extractable fraction from *Chlorokybus*, which can qualify as ‘pectin’. This fraction features uronic acids as well as a variety of neutral sugars. In particular, we describe here the structure of a novel aldobiouronic acid, β‐d‐GlcA‐(1→4)‐l‐Gal. Moreover, this fraction is made up of two distinct polymers, differing in their ionisation and degree of sulphation, and in their susceptibility to acid‐hydrolysis. Based on these data, we propose that *Chlorokybus* ‘pectin’ could be analogous to land plants’, presenting similar properties whilst being made of a unique set of polysaccharide structures. This would imply that some features of pectin were present in the earliest land plants and have structurally diverged, whilst keeping a similar organisation.

## AUTHOR CONTRIBUTIONS

Marie N. Rapin: Conceptualisation, investigation, writing ‐ original draft, review & editing.

Lorna Murray: NMR spectroscopy.

Ian H. Sadler: NMR spectroscopy, writing ‐ review & editing.

John H. Bothwell: Conceptualisation, supervision, funding acquisition, writing ‐ review & editing.

Stephen C. Fry: Conceptualisation, supervision, funding acquisition, writing ‐ original draft, review & editing.

## Supporting information


**Fig. S1:** Thin‐layer chromatography of acid‐hydrolysed polymer fractions from six species.
The TLC solvents were a, BAW; b, EPAW; both with two ascents. Each hydrolysate loading was derived from 15 μg of the polysaccharide, and each sugar in the marker mixtures (MM) was 3 μg. Polymer fractions were: P1 and P2, ‘pectins’; Ha and Hb, hemicelluloses a and b; W, mildly acidic wash after alkali; αC, residual ‘α‐cellulose’. Sugars in grey boxes and labelled in black are authentic pure monosaccharides (MM, marker mixture). Those labelled in blue are unknowns detected in the hydrolysates (named according to species and numbered according to increasing mobility; e.g. *Kf*3 is the 3rd‐fastest migrating spot from *Klebsormidium fluitans*). Those labelled in red are unusual sugars previously identified in some of the species tested: ‘GlcA‐Gal’ in the *Anthoceros* products is α‐d‐glucuronosyl‐(1→3)‐l‐galactose (Popper *et al*., 2003); 3MeGal in the *Chara* products is 3‐*O*‐methylgalactose (O'Rourke et al., 2015). ‘A1’ in the *Chlorokybus* products is shown in this work to be β‐d‐GlcA‐(1→4)‐l‐Gal.
**Fig. S2:** Thin‐layer chromatography of α‐amylase digestion products of AIR from six plant species.
After α‐amylase digestion, ethanol‐soluble products were analysed by TLC. Sample 1, 2 and 3 of each species are replicate amylase digests. TLC solvent: EPAW. Grey rectangle: marker mixture.
**Fig. S3:** NMR spectroscopy of aldobiouronic acid **A1** from *Chlorokybus* pectin.
2‐D gradient‐selected proton COSY NMR spectrum over the region 3.1–5.5 ppm. The disaccharide A1 is deduced to be β‐d‐glucuronosyl‐(1→4)‐l‐galactose. The residues are labelled A, α‐Gal; B, β‐Gal; C, β‐GlcA.
**Fig. S4:** Nuclear Overhauser effect (NOESY) ^1^H‐NMR spectrum and deduced structure of the novel aldobiouronic acid obtained from *Chlorokybus* pectin.
The residues are labelled A, α‐Gal; B, β‐Gal; C, β‐GlcA . X corresponds to the superimposed signals from A6a, A6b, B6a and B6b. Further details are in Table 1.
**Table S1:** Enzymes and conditions used to assay polysaccharide sugar contents
**Table S2:** Features of markers and of the main unknown compounds in both TLC solvent mixtures

## Data Availability

Data sharing is not applicable to this article as all new created data is already contained within this article and the supplementary material of this article.
