# Supplementary material for: Same but different — pseudo‐pectin in the charophytic alga Chlorokybus atmophyticus
Source: Physiol Plant. 2023 Nov 15;175(6):e14079. doi: 10.1111/ppl.14079 (PMC10953000; doi:10.1111/ppl.14079)
Supplement: Supplementary file 1 — Fig. S1: Thin‐layer chromatography of acid‐hydrolysed polymer fractions from six species. The TLC solvents were a, BAW; b, EPAW; both with two ascents. Each hydrolysate loading was derived from 15 μg of the polysaccharide, and each sugar in the marker mixtures (MM) was 3 μg. Polymer fractions were: P1 and P2, ‘pectins’; Ha and Hb, hemicelluloses a and b; W, mildly acidic wash after alkali; αC, residual ‘α‐cellulose’. Sugars in grey boxes and labelled in black are authentic pure monosaccharides (MM, marker mixture). Those labelled in blue are unknowns detected in the hydrolysates (named according to species and numbered according to increasing mobility; e.g. Kf3 is the 3rd‐fastest migrating spot from Klebsormidium fluitans). Those labelled in red are unusual sugars previously identified in some of the species tested: ‘GlcA‐Gal’ in the Anthoceros products is α‐d‐glucuronosyl‐(1→3)‐l‐galactose (Popper et al., 2003); 3MeGal in the Chara products is 3‐O‐methylgalactose (O'Rourke et al., 2015). ‘A1’ in the Chlorokybus products is shown in this work to be β‐d‐GlcA‐(1→4)‐l‐Gal. Fig. S2: Thin‐layer chromatography of α‐amylase digestion products of AIR from six plant species. After α‐amylase digestion, ethanol‐soluble products were analysed by TLC. Sample 1, 2 and 3 of each species are replicate amylase digests. TLC solvent: EPAW. Grey rectangle: marker mixture. Fig. S3: NMR spectroscopy of aldobiouronic acid A1 from Chlorokybus pectin. 2‐D gradient‐selected proton COSY NMR spectrum over the region 3.1–5.5 ppm. The disaccharide A1 is deduced to be β‐d‐glucuronosyl‐(1→4)‐l‐galactose. The residues are labelled A, α‐Gal; B, β‐Gal; C, β‐GlcA. Fig. S4: Nuclear Overhauser effect (NOESY) 1H‐NMR spectrum and deduced structure of the novel aldobiouronic acid obtained from Chlorokybus pectin. The residues are labelled A, α‐Gal; B, β‐Gal; C, β‐GlcA . X corresponds to the superimposed signals from A6a, A6b, B6a and B6b. Further details are in Table 1. Table S1: Enzymes and conditions use [file PPL-175-0-s001.pdf]

Physiologia Plantarum

Same but different — pseudo-pectin in the charophytic alga *Chlorokybus atmophyticus*

Marie N. Rapin,[1] Lorna Murray,[2] Ian H. Sadler,[2] John H. Bothwell,[3] Stephen C. Fry.[1*]

[1] *The Edinburgh Cell Wall Group, Institute of Molecular Plant Sciences, The University of Edinburgh, Daniel Rutherford Building, The King's Buildings, Max Born Crescent, Edinburgh EH9 3BF, UK*

[2] *EastChem School of Chemistry, The University of Edinburgh, The King's Buildings, Edinburgh EH9 3JJ, UK*

[3] *Department of Biosciences, Durham University, South Road, Durham, DH1 3LE, UK*

*Author for correspondence. Telephone +44 (0)131 650 6520, E-mail S.Fry@ed.ac.uk

Supplementary Information

Table S1: Enzymes and conditions used to assay polysaccharide sugar contents

Enzyme	Biological source	Commercial source*	Specific activity (U.mg^{-1})	Enzyme concentration (U. ml^{-1})	Substrate (mg.ml^{-1})	Buffer	Incubation temperature	Incubation time
Endo-1,4-β-xylanase	rumen microorganisms	Megazyme	~ 380	10	2.5	40 mM lutidine (acetate, pH 6.5)	20°C	16 h
Endo-1,5-α-arabinanase	*Aspergillus niger*	Megazyme	~ 10	5	2.5	170 mM acetic acid (pyridine, pH 4)	20°C	1 h
Endo-1,4-β-galactanase	*Aspergillus niger*	Megazyme	> 150	2.5	2.5	170 mM acetic acid (pyridine, pH 4)	20°C	1 h
α-Xylosidase	*'microbial'*	CPC Biotech	0.08 †	0.05	10	50 mM succinate (Na^+, pH 5.5)	40°C	30 min
Endopolygalacturonase	*Aspergillus aculeatus*	Megazyme	350	2.5	2.5	130 mM pyridine (acetate, pH 4.7)	20°C	1 h (Fig. 4)<br>16 h (Fig. 7)
β-Galactosidase	*Aspergillus niger*	Megazyme	~ 170	1	10	170 mM acetic acid (pyridine, pH 5)	20°C	48 h
α-Galactosidase	guar	Megazyme	~ 50	100	2.5	170 mM acetic acid (pyridine, pH 5)	20°C	48 h
Driselase (mixture of enzymes)	*Irpex lacteus*	Sigma	N/A	0.50%	10	130 mM pyridine (acetate, pH 4.7)	37°C	16–90 h
D-Galactose oxidase	*Dactylium dendroides*	Sigma	500	4	2.5	23 mM collidine (acetate, pH 6.0)	20°C	96 h

All the enzymes were freed of ammonium sulphate if present, and stock solutions were in made up in buffer; all the polysaccharide substrate solutions were in pure water.

*Commercial sources were Megazyme, Bray, Ireland (https://www.megazyme.com/); Sigma Chemical Co., Glasgow, UK (https://www.sigmaaldrich.com/GB/en); and CPC Biotech, Agrate Brianza, MI, Italy (https://www.cpcbiotech.it/en/).

† With maltose as the substrate.

Table S2: Features of markers and of the main unknown compounds in both TLC solvent mixtures

Sugar	R_{MeXyl} in BAW*	R_{MeXyl} in EPAW*	Staining with thymol	Staining same colour as… †
α-D-GlcA-(1→3)-L-Gal (in *Anthoceros*)	0.35	0.027	Pink/brown	
GalA	0.42	0.056	Pink/brown	
GlcA	0.46	0.074	Pink/brown	
Gal	0.61	0.41	Brownish	
Glc	0.68	0.49	Pink/brown	
Ara	0.69	0.63	Purple/blue	
Man	0.72	0.55	Pink/brown	
3-*O*-MeGal (in *Chara*)		0.73	Pink/brown	
Rib	0.77	0.78	Purple/blue	
Fuc	0.79	0.76	Pink/brown	
Xyl	0.85	0.77	Purple/blue	
Rha	0.96	0.92	Red/brown	
MeXyl	**(1)**	**(1)**	Purple/blue	
Chlorokybus cmpd **A1**	0.30			Hexose
*Ac*1	0.15			Hexose
*Ac*2	1.02	1.07		6-Deoxyhexose
*Ac*3	1.08	1.08		Pentose?
*Ca*1	0.53	0.35		Hexose
*Ca*2	0.96	0.98		6-Deoxyhexose
*Ca*3		1.02		Hexose
*Cs*1	0.12			Hexose
*Cs*2	0.31			Hexose
*Cs*3	0.99	1.051		Hexose
*Cv*1	0.13			Hexose
*Cv*2	0.36			Hexose
*Cv*3	0.47	0.19		Hexose
*Cv*4	0.52	0.30		Hexose
*Kf*1	1.00–1.04?	0.98		Pentose
*Kf*2		1.05		Hexose
*Kf*3		1.12		Pentose?
*Kf*4		1.17	Pink	
*Kf*5	1.13			6-Deoxyhexose
*Ul*1	0.50	0.096		Hexose
*Ul*2	1.01	1.07		6-Deoxyhexose

*R_{MeXyl} is the mobility on TLC relative to that of methyl β-xyloside (MeXyl) spot, the furthest migrating marker. Solvents were BAW and EPAW, each with two ascents on a plastic-backed silica-gel plate. Data for neighbouring sugars that were not reliably resolved are shaded in grey. Unknowns are named according to plant species and numbered according to increasing mobility; e.g. *Kf*3 is the 3rd-fastest-migrating spot from *Klebsormidium fluitans*. Blanks imply that the AIR-derived sugar was not visible in one solvent or the other, possibly because it co-migrated with a more prominent sugar.

† The staining colours are somewhat subjective; therefore, in the case of unknowns, the colour is reported as resembling a typical hexose (e.g. Glc but not Gal), a pentose (e.g. Xyl) or a typical 6-deoxyhexose [e.g. Rha (6-deoxy-Man) but not Fuc (6-deoxy-Gal)].

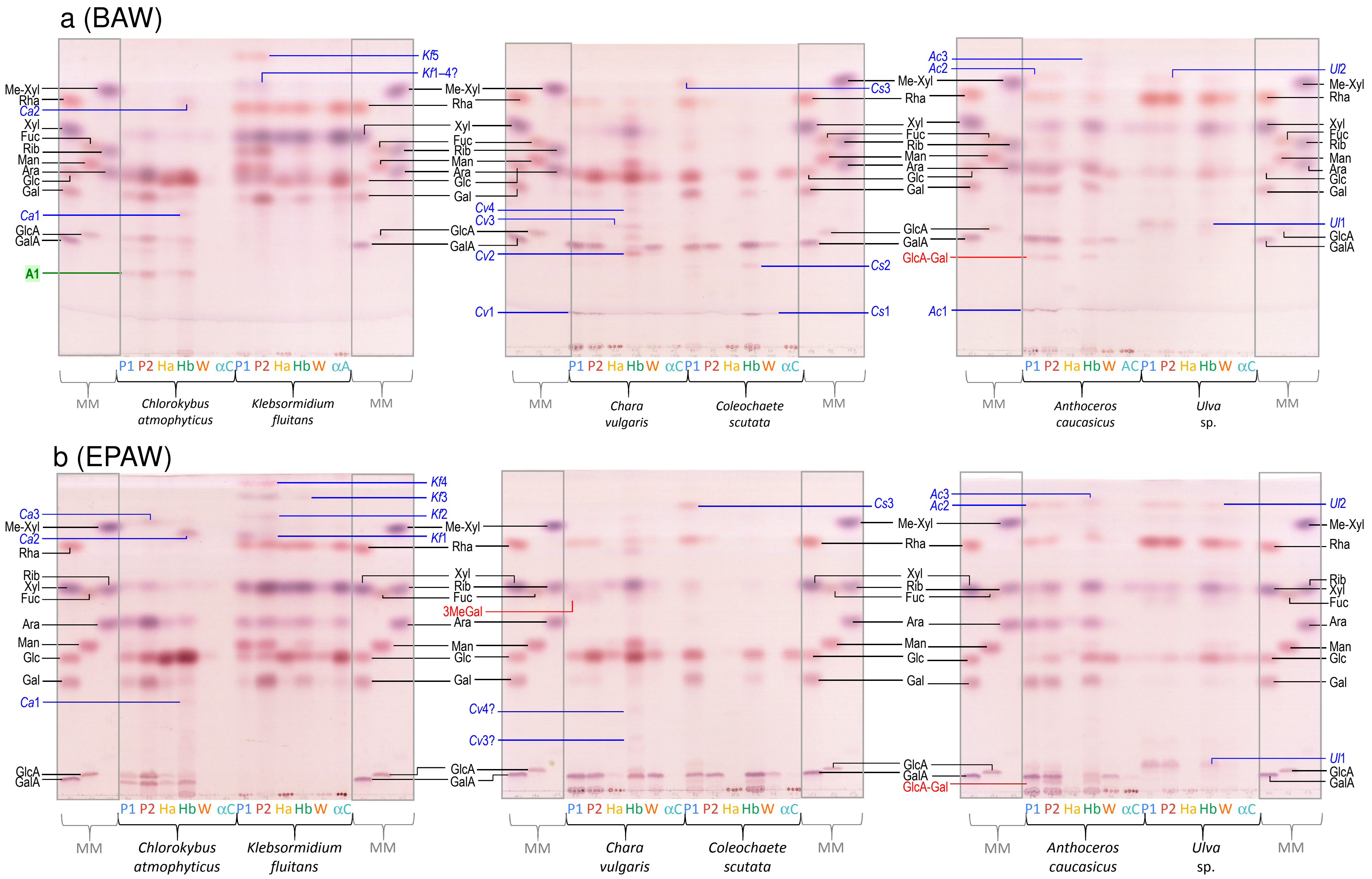

Fig. S1: Thin-layer chromatography of acid-hydrolysed polymer fractions from six species.

The TLC solvents were a, BAW; b, EPAW; both with two ascents. Each hydrolysate loading was derived from 15 µg of the polysaccharide, and each sugar in the marker mixtures (MM) was 3 µg. Polymer fractions were: P1 and P2, 'pectins'; Ha and Hb, hemicelluloses a and b; W, mildly acidic wash after alkali; αC, residual 'α-cellulose'. Sugars in grey boxes and labelled in black are authentic pure monosaccharides (MM, marker mixture). Those labelled in blue are unknowns detected in the hydrolysates (named according to species and numbered according to increasing mobility; e.g. *Kf*3 is the 3rd-fastest migrating spot from *Klebsormidium fluitans*). Those labelled in red are unusual sugars previously identified in some of the species tested: 'GlcA-Gal' in the *Anthoceros* products is α-D-glucuronosyl-(1→3)-L-galactose (Popper *et al.*, 2003); 3MeGal in the *Chara* products is 3-O-methylgalactose (O'Rourke et al., 2015). 'A1' in the *Chlorokybus* products is shown in this work to be β-D-GlcA-(1→4)-L-Gal.

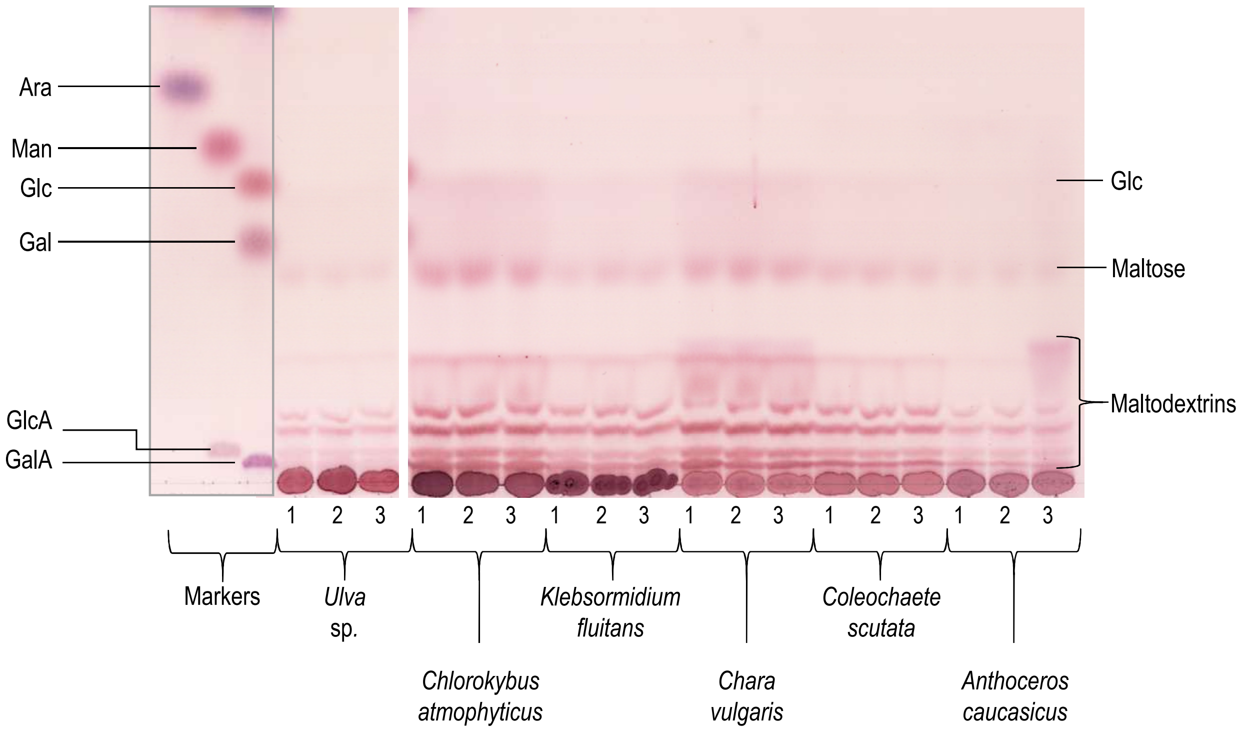

Fig. S2: Thin-layer chromatography of α-amylase digestion products of AIR from six plant species.

After α-amylase digestion, ethanol-soluble products were analysed by TLC. Sample 1, 2 and 3 of each species are replicate amylase digests. TLC solvent: EPAW. Grey rectangle: marker mixture.

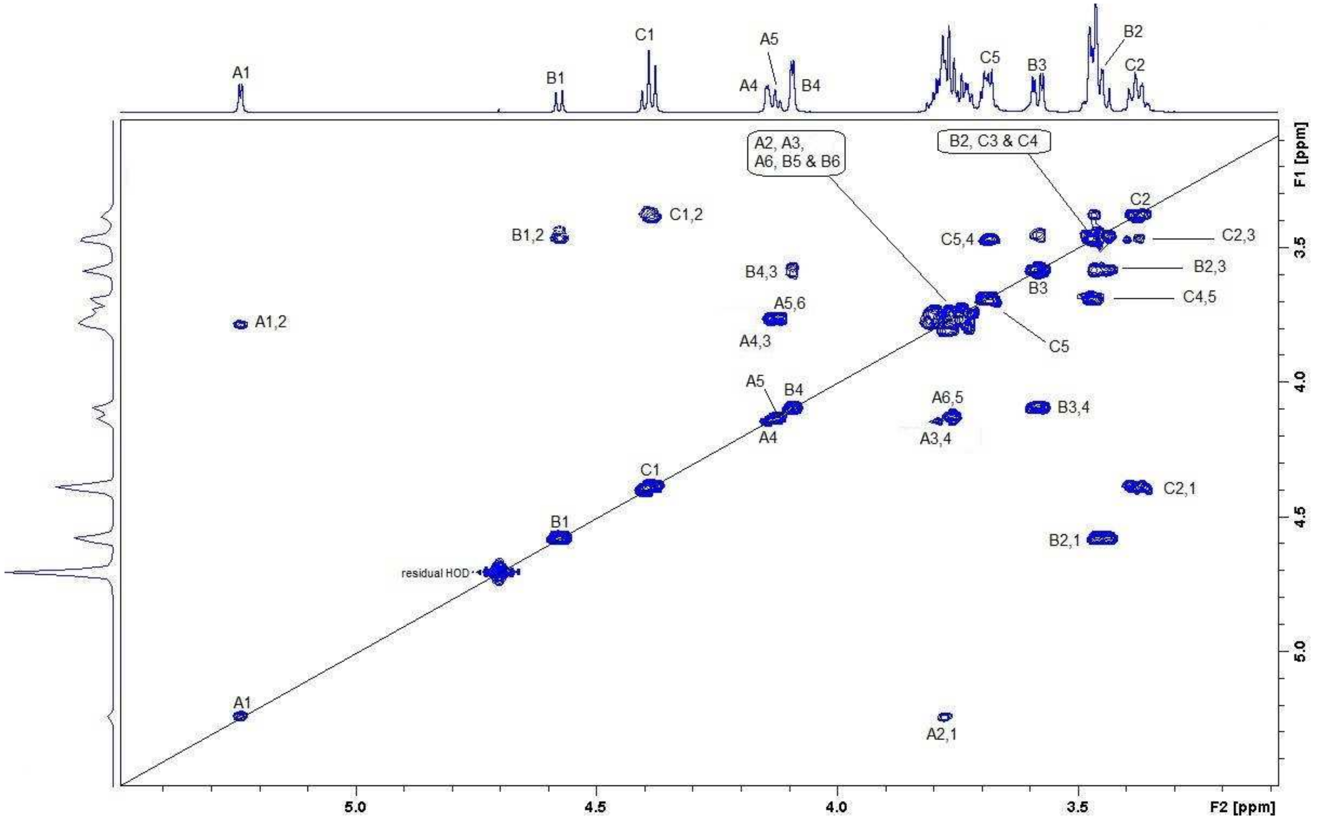

Fig. S3: NMR spectroscopy of aldobiouronic acid **A1** from *Chlorokybus* pectin.

2-D Gradient-selected proton COSY NMR spectrum over the region 3.1–5.5 ppm. The disaccharide A1 is deduced to be β-D-glucuronosyl-(1→4)-L-galactose. The residues are labelled A, α-Gal; B, β-Gal; C, β-GlcA.

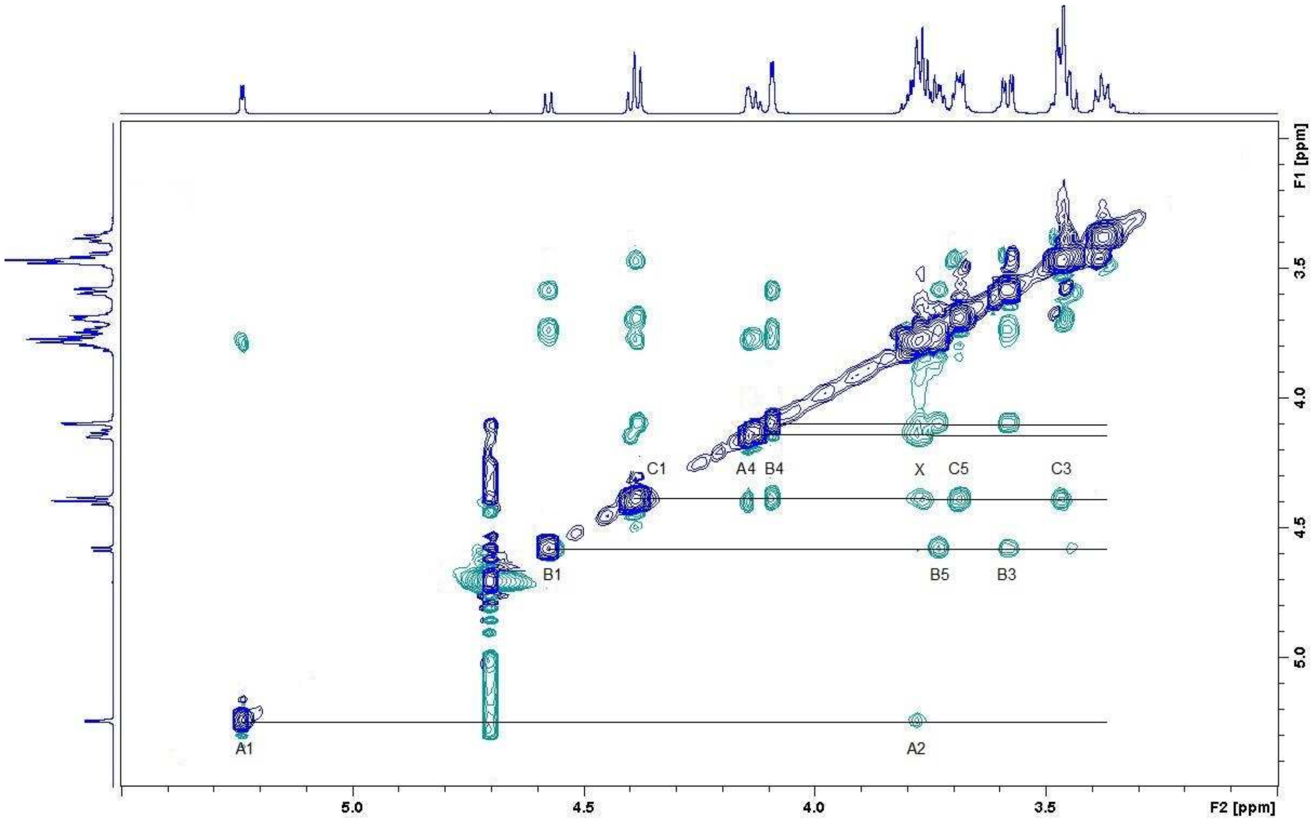

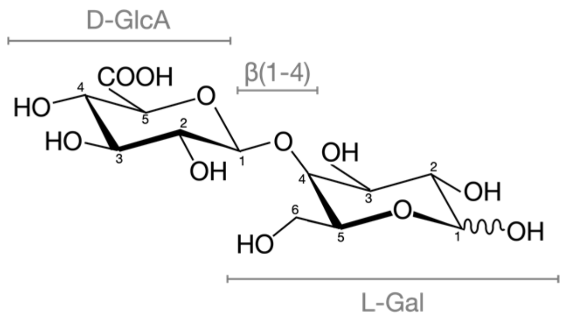

Fig. S4: Nuclear Overhauser effect (NOESY) ^{1}H-NMR spectrum and deduced structure of the novel aldobiouronic acid obtained from *Chlorokybus* pectin.

The residues are labelled A, α-Gal; B, β-Gal; C, β-GlcA . X corresponds to the superimposed signals from A6a, A6b, B6a and B6b. Further details are in Table 1.
